# Nonequilibrium Self-Assembly Time Forecasting by the Stochastic Landscape Method

**DOI:** 10.1021/acs.jpcb.3c01376

**Published:** 2023-07-05

**Authors:** Michael Faran, Gili Bisker

**Affiliations:** † Department of Biomedical Engineering, Faculty of Engineering, Tel Aviv University, Tel Aviv 69978, Israel; ‡ The Center for Physics and Chemistry of Living Systems, Tel Aviv University, Tel Aviv 6997801, Israel; § The Center for Nanoscience and Nanotechnology, Tel Aviv University, Tel Aviv 6997801, Israel; ∥ The Center for Light-Matter Interaction, Tel Aviv University, Tel Aviv 6997801, Israel

## Abstract

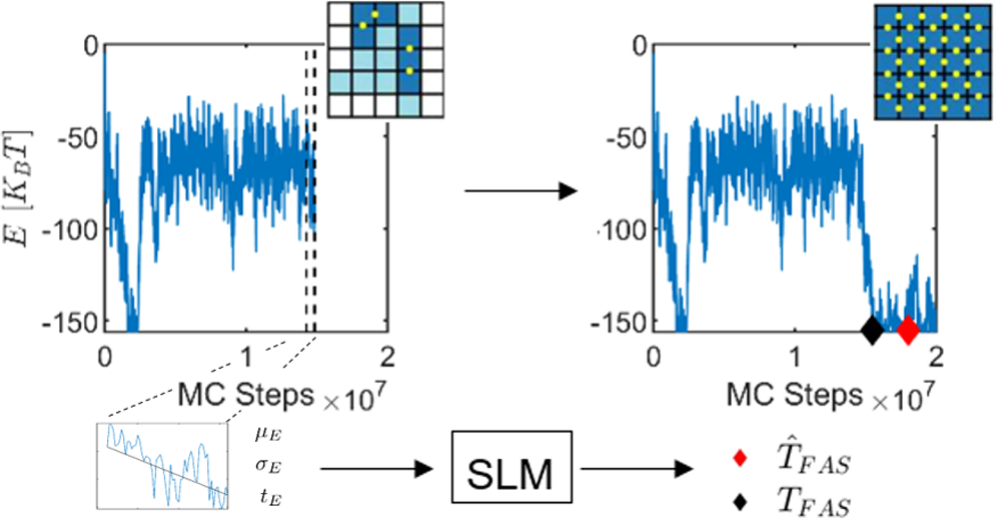

Many biological systems rely on the ability to self-assemble target structures from different molecular building blocks using nonequilibrium drives, stemming, for example, from chemical potential gradients. The complex interactions between the different components give rise to a rugged energy landscape with a plethora of local minima on the dynamic pathway to the target assembly. Exploring a toy physical model of multicomponents nonequilibrium self-assembly, we demonstrate that a segmented description of the system dynamics can be used to provide predictions of the first assembly times. We show that for a wide range of values of the nonequilibrium drive, a log-normal distribution emerges for the first assembly time statistics. Based on data segmentation by a Bayesian estimator of abrupt changes (BEAST), we further present a general data-based algorithmic scheme, namely, the stochastic landscape method (SLM), for assembly time predictions. We demonstrate that this scheme can be implemented for the first assembly time forecast during a nonequilibrium self-assembly process, with improved prediction power compared to a naïve guess based on the mean remaining time to the first assembly. Our results can be used to establish a general quantitative framework for nonequilibrium systems and to improve control protocols of nonequilibrium self-assembly processes.

## Introduction

Self-assembly is a process in which some form of overall order arises from local interactions between constituents of an initially disordered system. This key phenomenon arises in many physical, chemical, and biological systems.^1−8^ Specifically, living systems, at the molecular level, ubiquitously present an ability to self-assemble a variety of different structures emerging from a common set of building blocks.^9−12^ It is considered a fundamental process in intra-cellular activity, manifested, for instance, in molecular chaperones that actively participate in protein folding,^9,13,14^ the protein components of chromatin regulating gene expression,^15−17^ the spindle-assembly checkpoint in mitosis of eukaryotes,^18^ and the protofilaments that form microtubules.^19,20^ From a physical perspective, the aforementioned building-blocks diversity gives rise to a highly complex energy landscape, with many local minima corresponding to meta-stable states and kinetic traps, which may slow down the self-assembly process.^20−24^ To overcome this challenge, living systems utilize nonequilibrium driving, for example, by harvesting a chemical fuel to accelerate the self-healing of an “off-target” state into a desired target assembly.^19,25−27^ The advantages of a nonequilibrium drive have been demonstrated in experiments,^28−32^ simulations,^20,23,24,33,34^ and theoretical models.^35−37^ Examples include using KOH and 1,3-propanesultone as chemical fuels for self-healing in supramolecular chiral G-quadruplex hydrogels,^38^ using guanosine triphosphate (GTP) hydrolysis for self-healing the growing phase of microtubules,^39,40^ and the use of chemical fuel for kinetic proofreading^41,42^ in enzymes-substrates reactions,^43^ damaged DNA discrimination,^44^ and antigen discrimination,^45^ where preferred reaction pathways are executed using an active mechanism. Moreover, nonequilibrium self-assembly can be conceptualized as taking part in decision-making and molecular computation.^46−49^

Biomimicking the benefits of nonequilibrium drive has attracted great technological interest,^25^ with numerous demonstrations such as artificial light-harvesting systems,^50^ natural viral capsids,^50,51^ target-specific delivery of drugs and genes,^52,53^ formation of supramolecular hydrogels,^25,54−56^ colloidal diamond photonic crystals,^57^ and more.^10,11,21,58−60^ Realizing a nonequilibrium self-assembly system would further benefit from control protocols that could assist in navigating the system to the desired target while monitoring its state over time.^23,33,34,61−67^ Nevertheless, experimental realizations of these protocols are challenging and require quantitative, coarse-grained observables of the self-assembly process.^37,62,68^ The time to the first self-assembly can be viewed from the perspective of a mean-first passage time problem,^69−72^ where control protocols generally aim to reduce the assembly time. Previous works have examined this analogy to study first self-assembly times.^73−78^

Inspired by deterministic dynamical systems inference^79−81^ and correspondence between dissipation and structure,^82−88^ a quantitative inference of self-assembly is desired for predicting its outcome and optimizing the process using external control. The Bayesian Estimator of Abrupt change, Seasonality, and Trend (BEAST) algorithm^89^ is a general framework for detecting trends and abrupt changes in any time series data and providing change occurrence probability at any time point.^90^ This algorithm was originally utilized to detect human and natural disturbances based on the time series Landsat imagery.^91^ BEAST combines several models to decompose time series data, improving change-point detection accuracy with robust performance.^90^

Here, we lay the foundation for inference and prediction in nonequilibrium self-assembly processes. Using Monte Carlo (MC) simulations^20,21,68,92^ of a toy physical model,^24^ we realize a system of distinguishable interacting particles, where self-assembly targets are encoded through specific interactions between the monomers, such that the assembled structures are global energy minima of the simulated system. In analogy to different conformations adopted by proteins, each particle can adopt different internal states, which govern its interaction with other particles. A local, self-healing drive is included through the propensity of a particle to adopt the same internal state as its neighbors.^24^ We study the effect of the self-healing drive on the distribution of the time to the first assembly and show that it can be fitted by a log-normal distribution whose median and standard deviation decrease with increasing drive value. In order to provide a prediction for the time to the first assembly, we introduce a novel approach, termed the stochastic landscape method (SLM), which relies on the BEAST algorithm. We apply BEAST time series segmentation on the total energy of the system along the trajectories to extract the slow time-scale variation of the system from the segments. Then, this information is encoded in a map that relates the system’s slowly varying statistical moments and the remaining time to the first self-assembly event. We demonstrate the potential of the SLM approach to provide predictions for our model system for different drive values and show how the statistical moments of a transient segmented observable, such as the energy, can be utilized to predict nonequilibrium self-assembly times. Our method can be generalized to other nonequilibrium self-assembly systems and be used as a baseline for control protocol design.

## Self-Assembly Model

**Figure 1:**
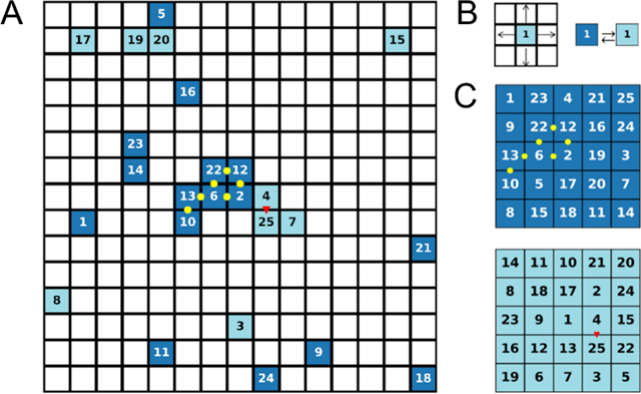
Model system Illustration. (A) Example of a lattice of size *L* = 15, occupied by *N* = 25 particles with *M*_T_ = 2 possible states identified by either a bright teal color or deep blue. The particles are distinguishable, denoted by numbers from 1 to 25. The red triangles and the yellow circles between some of the nearest-neighbor particle pairs denote a bond between two adjacent particles according to one of the encoded targets. (B) In each iteration, a physical move on the lattice (left) is attempted, followed by an internal state switch (right). The trial moves are accepted with a probability that depends on the energy difference and external drive, as described in the main text. (C) Two target structures in which all of the particles are in one of the two possible internal states. Each one has a unique spatial configuration of the *N* particles.

Our model^24^ consists of *N* distinguishable particles that can self-assemble *M*_T_ target structures. Each particle is represented by its labeled number *i* = 1, ···, *N* and internal state *s*_*i*_ = 1, ···, *M*_T_. The particles roam around a *L* × *L* square lattice (Figure 1A), translate to unoccupied adjacent tiles, change their internal state, and interact with neighboring particles (Figure 1B). Each tile can be either unoccupied or solely occupied by a single particle. A target structure of the self-assembly process is a unique spatial arrangement of the particle positions, given all of the particles adopt a specific internal state (Figure 1C). We assume that each internal state of the particles corresponds to a specific target, encoded by favored interactions between neighboring particles according to the target. These encoded targets are thus global minima in the energy landscape of the model. This model is motivated by molecular architectures, in which self-assembled structures rely on the specific conformation of the building blocks.^9,14,19,21^

When two particles, *i* and *j*, occupy adjacent lattice sites, they experience nearest-neighbor (n.n.) interaction, *J*(*s*_*i*_, *s*_*j*_), which depends on their internal states and the encoded targets. A particle pair *i*, *j* is defined as a neighboring pair if they are n.n. in one of the stored targets (Figure 1C). When two adjacent particles *i*, *j* on the lattice are a neighboring pair according to target *m*, and both share the internal state *s*_*i*_ = *s*_*j*_ = *m*, they experience a strong attraction, *J*_s_. If the pair *i*, *j* is not a neighboring one, the particles experience a weak interaction *J*_w_. Otherwise, if the particles are a neighboring pair according to target *m*, but only one of them has the appropriate internal state, *s*_*i*_ = *m*, *s*_*j*_ ≠ *m*, then the interaction strength is , and if none of them is at the internal state *m*, then the interaction strength is *J*_*w*_. Mathematically, the interaction energy between particles *i* and *j* can be written as
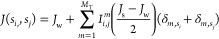
1where δ_*m*,*s*_*i*__ is the Kronecker delta and *I*_*i*,*j*_^*m*^ is a matrix representing the stored target, *I*_*i*,*j*_^*m*^ = 1 if the particles *i* and *j* are nearest neighbors according to target *m*, and zero otherwise.

The total energy of the system, *E*, is calculated by
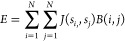
2where *B*(*i*, *j*) is the board nearest neighbors matrix, *B*(*i*, *j*) = 1 if particles *i* and *j* are nearest neighbors on the lattice according to the current state of the simulation, and zero otherwise.

The dynamics are simulated using a single-particle Monte Carlo Metropolis algorithm.^93^ Initially, the *N* particles are placed randomly on the lattice with *s*_*i*_ = 1 for *i* = 1, ···, *N*. In each iteration, a particle is randomly chosen and attempts to translate to an adjacent tile, either up, down, right, or left (Figure 1B), with periodic boundary conditions. If the destination tile is occupied, the move is rejected. Otherwise, the total energy difference Δ*E* between the post- and pretranslation configurations is calculated, and the probability *p* to accept the suggested move is given by^93^
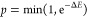
3The energy in eq 3 and generally throughout this paper is given in units of *K*_B_*T* (Table 1).

**Table 1:** Simulation Parameters

parameter	notation	value
number of particles	*N*	25
grid size	*L*	15
number of targets	*M*_T_	2
strong energy	*J*_s_	–4 *K*_B_*T*
weak energy	*J*_w_	–1 *K*_B_*T*
external driving	Δμ	[0.6, 0.8, 1.0, ···, 2.8] *K*_B_*T*
simulation time cap	*T*	5 × 10^7^

After each attempted physical move, a particle is chosen at random again, along with a new random internal state, different from its current one (Figure 1B). Under equilibrium conditions, the energy difference is calculated, and the internal state switch is accepted with probability, according to eq 3.

When a self-healing drive is introduced, a particle is more likely to adopt the internal state of its neighbors and less likely to change its internal state if it is similar to its neighbors. Specifically, if the chosen particle for an internal state switch has two or more n.n. with the same internal state, the acceptance probability of an attempted move to switch its internal state to that of its neighbors increases by Δμ. Moreover, if initially, the internal state of the chosen particle is the same as two or more of its n.n., the acceptance probability of an attempted move to switch its internal state to a different one decreases by Δμ. Therefore, the acceptance probability *q* of an internal state switch under external driving is given by^24^
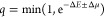
4where the + or – signs in the argument of the exponent are chosen according to the rule defined above, depending on whether the state switch is favored or not. These modified dynamics break detailed balance.^93^

The values of the parameters of the simulations, summarized in Table 1, were chosen based on an initial analysis of the system performance at equilibrium and the available computational resources (see Model Analysis Section in the Supporting Information). We note that the single-particle Monte Carlo simulation may not fully capture all quantitative features of realistic dynamics since collective clusters diffusion is suppressed;^94,95^ however, it can still provide insights into the process of self-assembly.^96−99^

## Results and Discussion

### MC Simulations

We begin by analyzing the self-assembly process under equilibrium conditions, Δμ = 0, quantified by the time to the first assembly, *T*_FAS_, starting from random initial conditions, and the total time spent at the target state, *T*_target_, when starting in one of the stored targets, as a proxy for target stability (see Model Analysis Section in the Supporting Information). In agreement with previous findings,^24^ we find a fundamental trade-off in equilibrium self-assembly, where target stability can come at the cost of the assembly speed (Figure S1). We, therefore, set to explore the self-assembly performance under an external drive.

**Figure 2:**
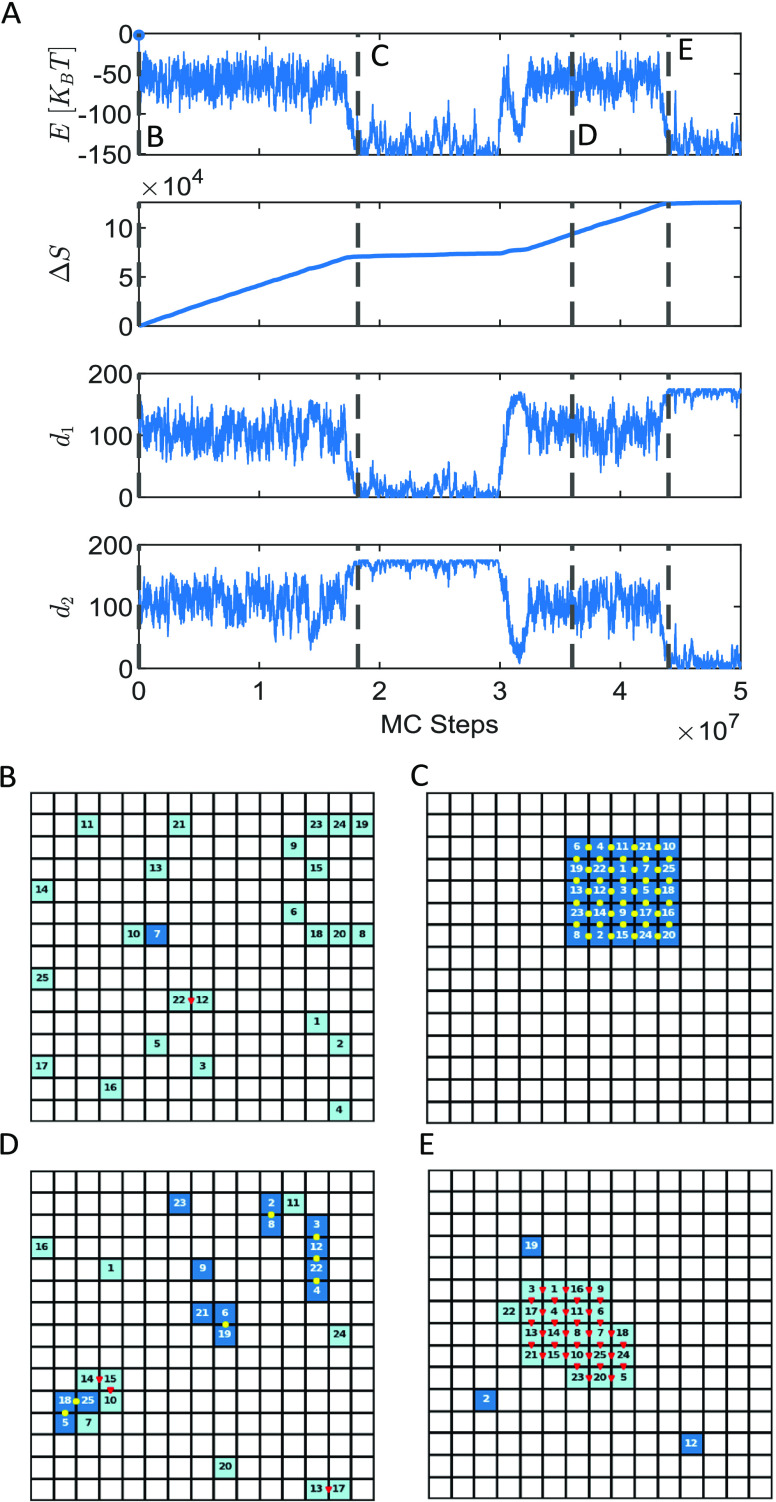
Simulation trajectories. (A) Energy *E*, total entropy production Δ*S*, and the distance from the two stored targets, *d*_1_ and *d*_2_, are plotted as a function of the MC steps. The vertical dashed lines indicate selected time points along the realization, corresponding to (B) initial conditions, (C) the first self-assembly event of target 1, (D) a potential kinetic trap characterized by energy fluctuations around a value higher than the global minima, and (E) approaching the successful self-assembly of target 2. The corresponding lattice states are plotted.

We simulated the system using the parameters listed in Table 1, with 1000 realizations for each value of the drive (See Numerical Implementation Section in the Supporting Information). During the trajectory of 5 × 10^7^ MC steps, we record the total energy *E* of the system, the total entropy production, Δ*S*, and the distance to the two targets, *d* (Figures 2A and S2). Δ*S* is calculated by summing up the log ratio of the probability of an accepted move and the probability of its reverse.^85,100^ The distance to target *m*, namely, *d*_*m*_, is calculated for each target separately by comparing the particles’ current adjacency matrix and the adjacency matrix of the two targets (see Distance from Target Calculation Section in the Supporting Information). Whenever *d*_*m*_ = 0 or *E* = −160 *K*_B_*T* (the global minimum energy value of the system), the corresponding target *m* is assembled. Snapshots of the particle positions and internal states along a single trajectory can be seen in Figure 2B–E and Movies S1, S2, and S3.

We observe that a successful assembly of a target structure is accompanied by a sharp change in the slope of the total entropy production Δ*S* (Figure 2C). While the system remains close to the assembled target, manifested in small values of *d*_*m*_, the entropy production rate decreases, which can be attributed to decreased fluctuations due to the emergence of a stable order in the system. Moreover, the trajectory includes segments of energy and distance fluctuations around values that do not correspond to one of the assembled targets, which we attribute to kinetic traps giving rise to meta-stable states in the dynamics.

### First Self-Assembly Time Statistics

**Figure 3:**
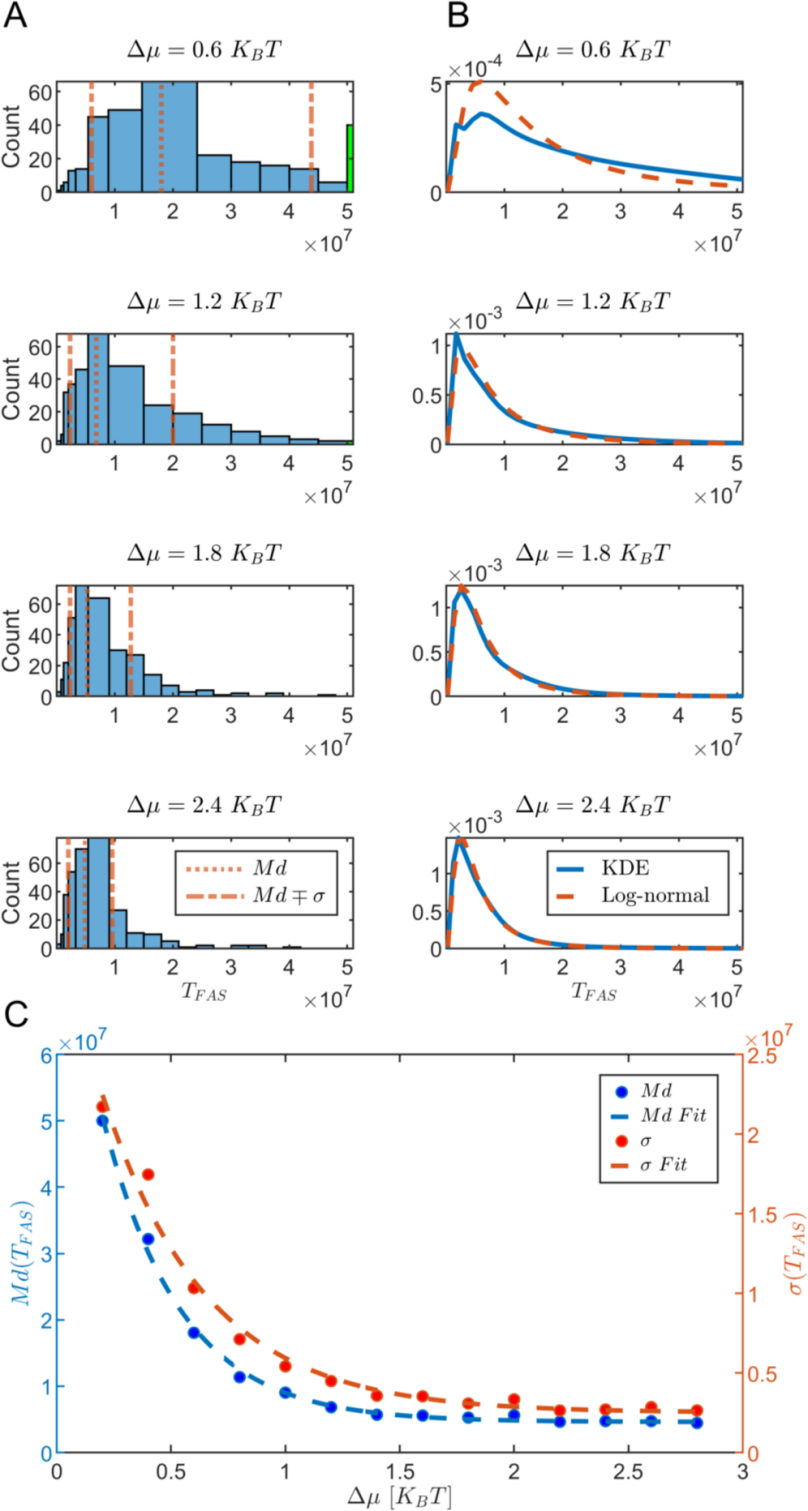
Simulation result statistics. (A) Simulation realizations histograms of the time to the first assembly for nonequilibrium drives, Δμ = 0.6, 1.2, 1.8, and 2.4 *K*_B_*T*. The dotted vertical lines are the median values, Md, of the histogram, and the dashed lines are the 16th and 84th percentiles of the data, respectively, corresponding to the standard deviation, σ. The green bins stand for the realizations in which no assembly occurred. (B) Fit for the simulations realization histograms with a kernel density estimator (KDE, blue) and a log-normal distribution (dashed orange). (C) Median Md (blue), and the standard deviation σ (red) of the time to the first assembly histograms as a function of the drive value, Δμ, with an exponential fit, *A*e^–*b*Δμ^ + *C* (dashed curves).

During a realization in which an assembly occurs, we record the first self-assembly time (in MC steps), *T*_FAS_. Histograms of the *T*_FAS_ for several values of the drive can be seen in Figure 3A, where we used unequal binning to capture the underlying distribution of the data. For values below the median of the data, we chose equal binning in the log scale, whereas for values above the median, we used the standard Scott’s rule for binning.^101^ Finally, all of the realizations in which an assembly did not occur were captured in a single bin corresponding to the total number of MC steps of the simulation.

The data were fitted by a Kernel density estimator (KDE) with a positive support^102^ and a log-normal distribution (Figure 3B). As the drive value increases, the fit of the log-normal distribution better captures the data. We note that for low values of the drive, the probability of not observing a successful assembly during the realization increases, so the simulation results cannot provide the full distribution of *T*_FAS_.

Owing to the simulations in which there is no assembly, the mean time to the first self-assembly would be an underestimation of the true value. Therefore, we focus on the median value, Md (dotted orange line in Figure 3A). In addition, we label one standard deviation, σ, above and below the median in the log scale, corresponding to the 16th and 84th percentiles (dashed orange lines in Figure 3A). Histograms and distributions of *T*_FAS_ for equilibrium and for additional drive values appear in Figure S3 (see Additional MC Simulation Results Section in the Supporting Information).

Both the median, Md, and the standard deviation, σ, decrease with increasing the drive down to a finite positive value (Figure 3C). The trend could be captured by an exponential decay, and the data were fitted using *A*e^–*b*Δμ^ + *C*. The fit parameters are listed in Tables S1 and S2. The finite positive value of the median of *T*_FAS_ for large drive values stems from the minimal time required for the diffusion of the particles. The system must explore its phase space, seeking one of the global minima, where all of the particles are in their desired locations according to one of the encoded targets.

### Remaining Time to the First Assembly

The remaining time to the first assembly, *t*_*r*_, measured in MC steps, is defined as the difference between the time to the first assembly, *T*_FAS_, for the trajectory at hand, and the observation time, *t*, i.e., *t*_*r*_ = *T*_FAS_ – *t*, where 0 ≤ *t* ≤ *T*_FAS_. Given the log-normal distribution of *T*_FAS_, we examined the distribution of *t*_*r*_, keeping in mind that *t*_*r*_ = *T*_FAS_ at *t* = 0. Indeed, we find that the *t*_*r*_ distribution can be approximated as a log-normal distribution as well (Figure S4), so its logarithm is fully characterized by the first two moments (see Remaining Time to First Assembly Statistics Section in the Supporting Information). Therefore, the mean and error of the prediction of log(*t*_*r*_) provide an assessment of the predictive power of our approach. As a result, we aim to provide predictions *Ŷ* for the remaining time to the first assembly in log scale *Y* = log(*t*_*r*_).

For the prediction, we rely on a segmented and down-sampled trajectory observable, *e.g.*, the energy, *E*. Without additional analysis, a naïve guess *Ŷ* for *Y* would be the mean value regardless of the statistical characterization of the observable. Here, we seek to utilize the statistical moments of the energy, *E*, to improve the prediction power of the remaining time to the first assembly, *t*_*r*_.

### Stochastic Landscape Method

The Stochastic Landscape Method (SLM) is an empirical approach for improving the forecast of the remaining time to the first self-assembly along a realization, using the information of the simulated macro-parameters. The key idea is to segment the trajectory data using the BEAST algorithm (see The BEAST Algorithm Details Section in the Supporting Information) and exploit the statistical information of the observable in each segment to predict the remaining time until a self-assembly event, better than the naïve guess based on the mean value of *t*_*r*_. The SLM can be considered as a supervised machine learning algorithm, as it uses input and output pairs to provide a prediction for unseen data,^103^ where the input data are the statistical information of the trajectory segments, and the output is the remaining time to the first assembly. In the following section, we explain the physical intuition behind the choice of the input data, which can also grant the SLM virtue of interpretability.

#### Motivation

We seek to divide the trajectory of an observable into temporal segments. Here, we chose the energy of the system as the parameter to segment. In our system, the combinatorial interplay of the interaction between the different particles results in a rugged energy landscape due to the kinetic traps in numerous local minima. An example of the simulation dynamics can be seen in the Movies S1–S3, where the trajectory can be considered as “jumping” between kinetic traps.

Along the trajectory, the energy of the system fluctuates with characteristic mean and standard deviation values at each of the meta-stable kinetic traps. When jumping to another kinetic trap, the temporal behavior of the energy changes to new mean and standard deviation values. Occasionally, the system spends time in transition states between two kinetic traps, where the energy value shows a nonzero trend while drifting to lower energy values or, rarely,^104^ to higher energy values. Therefore, we assume that each segment can be associated with a different “macrostate” in the complex rugged energy landscape of the system,^105−109^ such that the statistics within each segment is a characterization of the position on the energy landscape. We thus describe each segment by its statistical features:^110^ the mean, standard deviation, and the mean trend of the segment,^89^ which are referred to as the “stochastic coordinates,” and we assume they are drawn from a different probability distribution in the different segments.^109^

**Figure 4:**
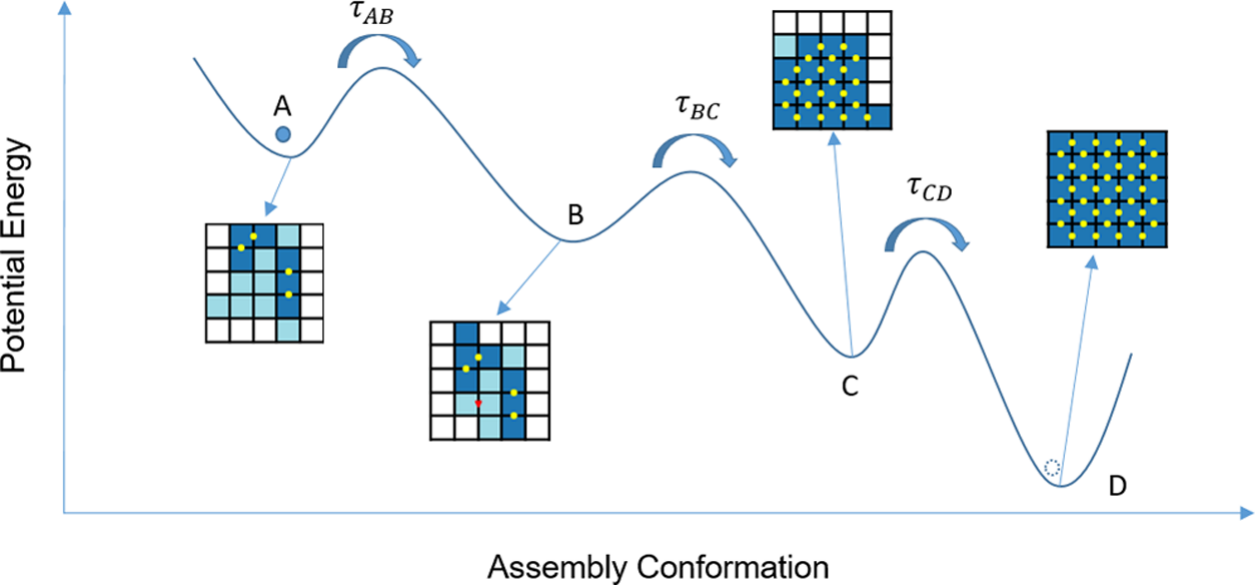
Self-assembly and Kramer’s escape rate illustration. The self-assembly process has a rugged energy landscape, and the system needs to cross multiple barriers between local minima (A–C) on the path to the target structure (D). Each barrier is associated with a typical escape rate, so the time to the first assembly can be approximated by the sum of the dwell times in the local minima, *T*_FAS_ ≈ τ_AB_ + τ_BC_ + τ_CD_, in this case.

We now refer to the remaining time to self-assembly prediction. Although not every segment is necessarily meta-stable, the time spent in meta-stable states significantly contributes to the time to the first assembly *T*_FAS_, as indicated by the concept of “low rattling.”^48^ According to Kramer’s escape rate theory,^111^ the average transition time between two meta-stable states in the energy landscape is determined by the barrier height, the energies of the two states, and the corresponding fluctuations. Inspired by Kramer’s idea, we expect that the time to the first assembly *T*_FAS_ is correlated to the sum of the transition times τ_*k*_ between meta-stable states visited along the system path to assembly, so *T*_FAS_ ≈ ∑_*k*_τ_*k*_. (see Figure 4). Following this meta-stability picture, we hypothesize that the information inferred by the stochastic coordinates is valuable for *t*_*r*_ prediction. Furthermore, if the system is found in a transition segment with a negative trend of the energy value, we anticipate the system is moving closer to its global minima, which is one of the target structures. Therefore, we expect the stochastic coordinates to carry valuable information for *t*_*r*_ prediction.

#### BEAST Algorithm

The SLM relies on an automated division of a trajectory observable into segments with different statistics. The BEAST algorithm was chosen for this purpose due to its versatile utilization and robustness.^89^ Moreover, it does not require specifying the number of anticipated statistical change points in the data, but rather, only a maximal number of change points is needed. BEAST assesses the probability of the number of statistical changes in the data and, by default, chooses the median number of this distribution.

The BEAST algorithm assumes a given time series is constructed from intervals of varying duration, each with Gaussian noise. The underlying unknown model is characterized by a set of model parameters, such as the periods of the seasonal signals, the number of seasonal and trend change points and their timings, the parameters of a linear trend and harmonic model of seasonality, and the magnitude of the Gaussian noise of each segment. BEAST aims to find the model parameters that would yield the time series statistics based on Bayesian inference by maximizing the posterior probability distribution, which is the product of a likelihood and the prior distribution of the model parameters, according to Bayes’ theorem. Since the posterior distribution is analytically intractable, the algorithm uses a Monte Carlo Markov Chain for sampling this distribution, to be used for the posterior inference.^89,112,113^

While BEAST can detect both trend changes and seasonal change points, here we focus on segmenting the data only according to trend changes, as there is no reason for seasonality in our case. Moreover, a minimum duration for each segment or a maximal number of change points can be defined in order to coarse-grain short-lived states. In our case, the minimum duration was set to one percent of the total simulation time (see SLM Implementation on Nonequilibrium Self-Assembly Section in the Supporting Information).

**Figure 5:**
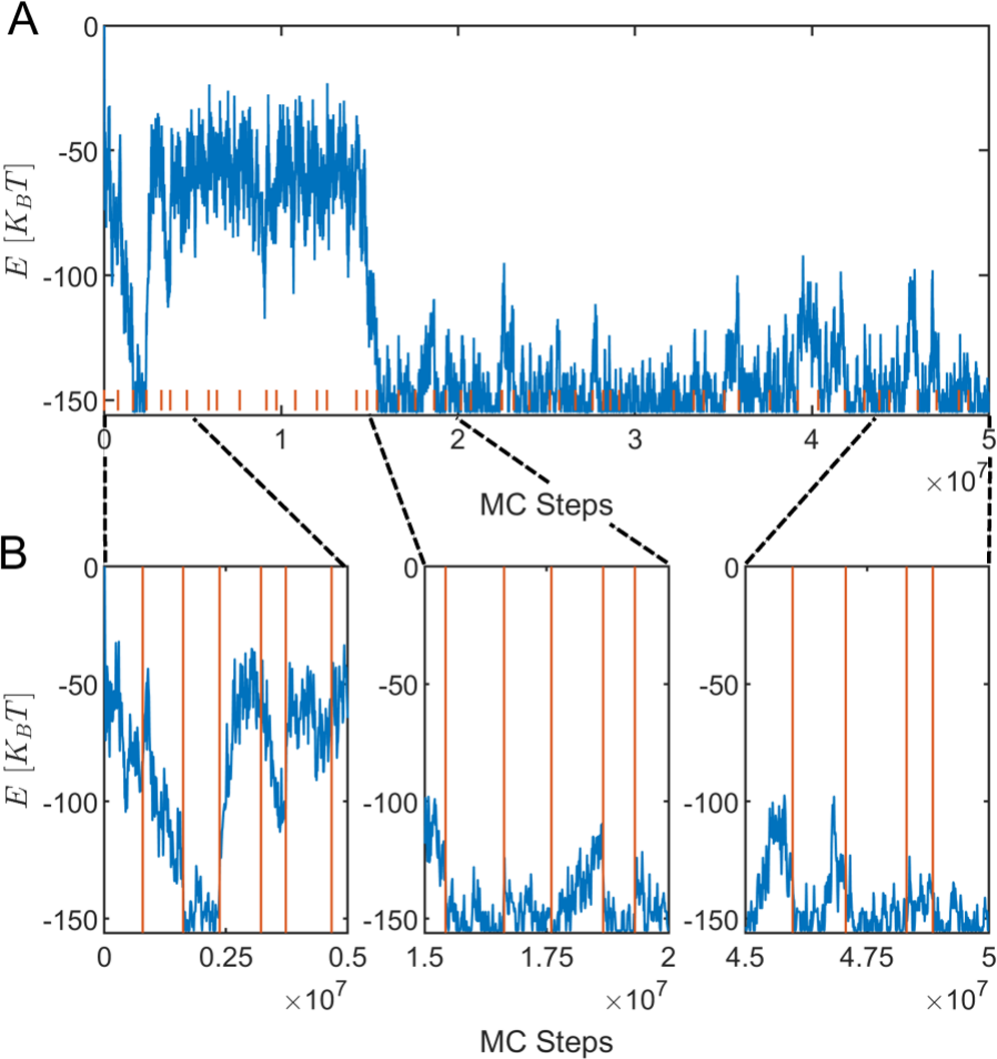
BEAST Segmentation. (A) Energy *E* of the system as a function of the MC steps for a realization of the system under nonequilibrium drive. The parameters are listed in Table 1, with Δμ = 1. The minimum value of the energy corresponds to a successful target assembly. The vertical orange lines indicate the change points in the statistics detected by the BEAST algorithm with a minimal segment–duration constraint of one percent of the total simulation time. Each segment between two change points is considered a “macrostate.” (B) Zoom-in on three different windows of the trajectory. Vertical orange lines mark the corresponding change points.

An example of the segmentation of an energy trajectory according to the BEAST algorithm can be seen in Figure 5A. Zooming into several segments, we can see that the BEAST algorithm captures significant changes in the signal statistics (Figure 5B). More examples of segmented energy trajectories are presented in Figure S5.

#### SLM Flowchart

**Figure 6:**
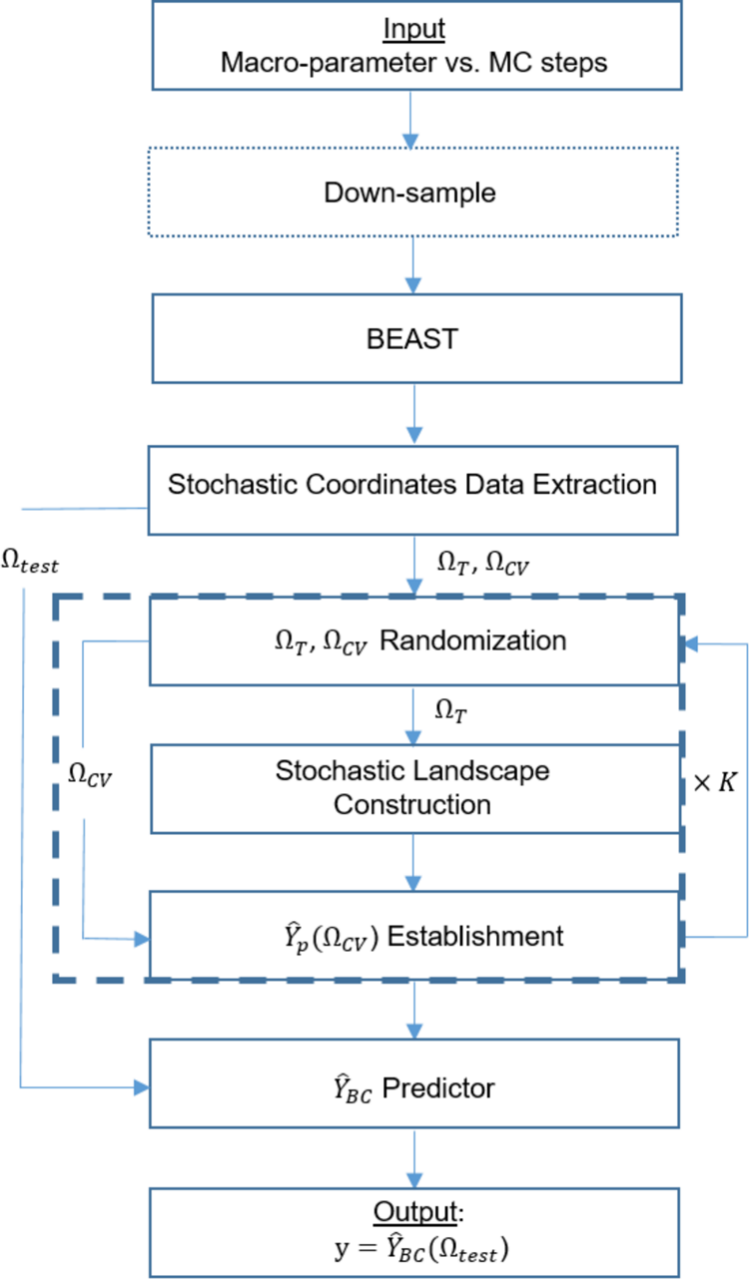
Flowchart of the SLM prediction process. The different steps are described in the text. The dotted rectangle is an optional step, and the dashed frame represents the repeated steps for cross-validation bias correction.

The SLM is an end-to-end algorithm developed for this work in order to infer the emerging complex dynamics of nonequilibrium self-assembly, specifically predicting the statistics of the remaining time to the first assembly, *t*_*r*_. Nevertheless, it is a general framework that can be modified and applied to other complex stochastic systems. The general step-by-step flowchart of the SLM is depicted in Figure 6, where the specific technical and detailed implementation of the method on the nonequilibrium self-assembly process can be found in the Supporting Information.

As a first step, the input of the SLM is a set of macro-parameter trajectories realized with the same parameters from random initial conditions, where a larger number of trajectories improves the statistics. Second, due to computation limitations, the trajectories are down-sampled to effectively limit the minimum duration of each segment. This step depends on the task in question and is generally optional. Third, we use the BEAST algorithm to segment the data with specified conditions (See Figures 5 and S5), i.e., a minimum duration of a segment is one percent of the total simulation time in our case. Fourth, the segments before the first self-assembly, Ω_FAS_, are collected, as we aim to provide a prediction for the remaining time to the first assembly. This step is also optional and depends on the question at hand. Then, the stochastic coordinates (mean, standard deviation, and the mean trend) are calculated for the chosen segments, Ω_FAS_, and each segment is labeled with the logarithm of its corresponding remaining time to the first assembly, *Y* = log(*t*_*r*_), based on the log-normal distribution of *t*_*r*_. The stochastic coordinates are normalized by subtracting their respective mean values and dividing them by their standard deviation, where the normalization is done for all of the segments in Ω_FAS_ together. A principal component analysis (PCA) is applied to the stochastic coordinates, and the first two principal components are chosen for the following steps.

Fifth, the segments Ω_FAS_ are randomly distributed between three sets: the training set, Ω_T_, the cross-validation set, Ω_CV_, and the test set Ω_test_. Sixth, the stochastic landscape is constructed from Ω_T_ following the stochastic coordinate results in the set, by a 2-D triangulation and data smoothing of the *Y* = log(*t*_*r*_) values as a function of the two principal components (see Fig. S6 for a visualization). Seventh, we use the stochastic landscape for generating a *Y* = log(*t*_*r*_) prediction for the cross-validation (CV) set Ω_CV_, which is referred to as the primary predictor *Ŷ*_*p*_(Ω_CV_). The fifth to seventh steps are repeated *K* = 10 times to get *K* different *Ŷ*_*p*_ primary predictors, and the bias between each of the predictors *Ŷ*_*p*_ and their true values *Y* is calculated for the segments in the CV set (Fig. S7). Eighth, we construct the stochastic landscape one last time for all of the data in the training and cross-validations sets, Ω_CV_ ∪ Ω_*T*_, and subtract the calculated bias, resulting in a biased corrected predictor *Ŷ*_BC_. Finally, *Ŷ*_BC_ provides a prediction on the test, and the output of the procedure is ***y*** = *Ŷ*_BC_(Ω_test_).

#### SLM Performance Evaluation

We quantify the performance of the SLM in three ways: (1) the correlation between the true value, *Y*, and the predicted values, *Ŷ*_BC_, (2) the mean error of the predicted values *Ŷ*_BC_ compared to a naïve guess based on the mean value *Ŷ*_*M*_ of the remaining time to the first assembly in a log scale, and (3) the Kullback–Leibler Divergence (KLD) between the Δ*Ŷ*_BC_ distribution and the data distribution Δ*Y*_test_, which is compared to the KLD between the Δ*Ŷ*_M_ distribution and Δ*Y*_test_, all defined below.

The mean predictor *Ŷ*_M_ is the mean of the log(*t*_*r*_) values for all of the segments in the set Ω_CV_ ∪ Ω_T_. This is the naïve guess if no other information is available. We expect the SLM predictor *Ŷ*_BC_ to perform better than the mean predictor *Ŷ*_M_, manifested in a smaller prediction error. To define the data distribution Δ*Y*_test_, we first bin the data according to the values of *Ŷ*_BC_. For each bin, we get a distribution of the true values, i.e.,, the remaining time to the first assembly for the test set in a log scale, *Y*_test_, subtracted from their mean, Δ*Y*_test_ = ⟨*Y*_test_⟩ – *Y*_test_. Similarly, we define the distributions Δ*Ŷ*_M_ = *Ŷ*_*M*_ – *Y*_test_ and Δ*Ŷ*_*BC*_ = *Ŷ*_BC_ – *Y*_test_ for each bin. We also calculate the relative weight of each bin by the ratio of the number of data points in the bin to the total number of data points in *Y*_test_. The distributions of Δ*Ŷ*_BC_ and Δ*Ŷ*_test_ are evaluated by the kernel density estimation (KDE)^102^ (see the distributions for Δμ = 1.6 in Figure S8 in the Supporting Information). Then, we compare the KLD between Δ*Ŷ*_BC_ and Δ*Ŷ*_test_, to the KLD between Δ*Ŷ*_M_ and Δ*Ŷ*_test_, for each bin, and perform a weighted average according to the weight of the bins to yield KLD_BC_ and KLD_M_, respectively. The KLD between two probability distributions, *f*_1_(*x*) and *f*_2_(*x*) is^114^
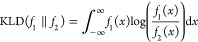
5As the KLD is a measure of the difference between two probability distributions, smaller KLD values indicate a better estimator of the true values. These evaluations were applied to the simulation data with the parameters listed in Table 1, and they are presented along with the prediction results in the next section.

### Self-Assembly Remaining Time Predictions

**Figure 7:**
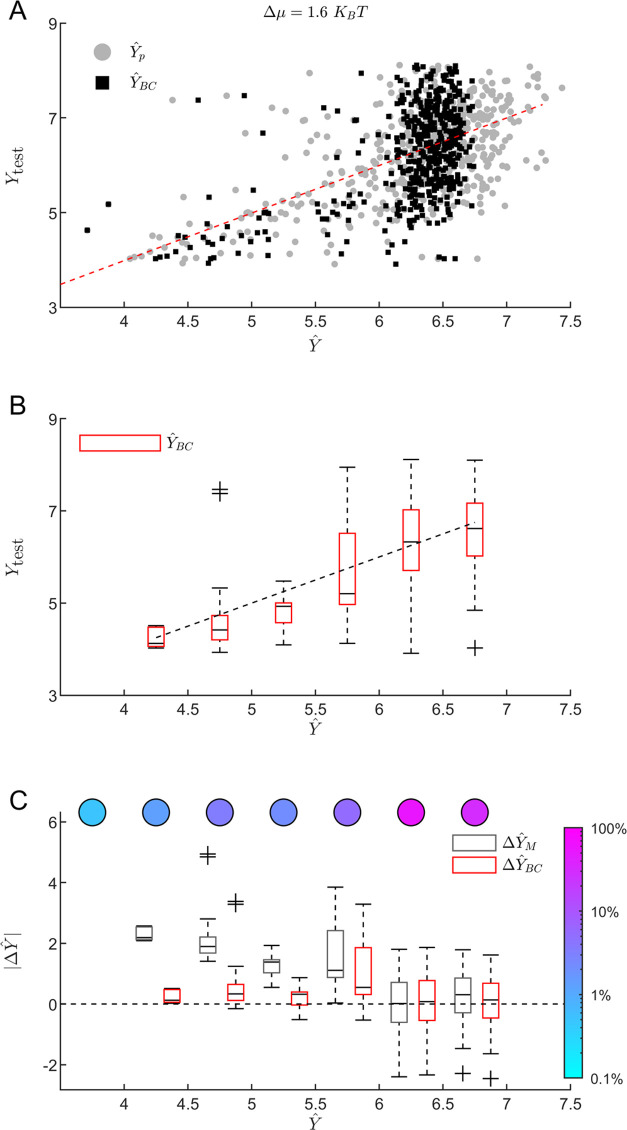
Results of the SLM prediction for the remaining time to the first assembly for the test set, for Δμ = 1.6 *K*_B_*T*. (A) *Y*_test_ results are shown *vs* their predicted values *Ŷ*, before the bias correction (*Ŷ*_p_, gray circles) and after the bias correction (*Ŷ*_BC_, black squares). The red line shows the linear regression of the data. (B) Box plot of the *Y*_test_*vs* the calculated *Ŷ*_BC_, where the data are binned according to the *Ŷ*_BC_ values (see main text).The box plot whiskers represent the standard interquartile range (IQR), and outliers are defined to be above 1.5 IQR (plus markers). The dashed unit slope, linear line (*y* = *Y*_test_) represents the results of a perfect predictor. (C) Absolute values of Δ*Ŷ*_M_ (black) and Δ*Ŷ*_BC_ (red) *vs* the respective predictor value *Ŷ* for each bin. The dashed black line at Δ*Ŷ* = 0 represents a perfect predictor with zero error. The color bar indicates the relative weight of the data for each bin, calculated from the relative number of data points. Bins with weights less than 1% are omitted.

The results of our test set prediction before and after the bias correction, *Ŷ*_p_ and *Ŷ*_BC_, for the remaining time to the first assembly, are presented in Figure 7A for the drive Δμ = 1.6 *K*_B_*T*, and in Figures S9A, S10A, and S11A, for drive values Δμ = 1, 2.2, and 2.8 *K*_B_*T*, respectively. The figures present sample-wise scatter plots of the remaining time to the first assembly for the test set, *Y*_test_, *vs* the predicted values, *Ŷ*_BC_. The Pearson correlation coefficient between *Y*_test_ and the *Ŷ*_BC_ was found to be in the range between *R* = 0.34 and *R* = 0.52 (*p* < 0.01) for all of the drive values (see Table 2).

**Table 2:** Performance Evaluation of the SLM

Δμ	*R*	KLD_M_	KLD_BC_
0.6	0.34	0.27	0.07
0.8	0.4	0.7	0.07
1.0	0.52	1.02	0.06
1.2	0.4	0.64	0.12
1.4	0.43	0.77	0.07
1.6	0.5	1.36	0.18
1.8	0.52	1.29	0.32
2.0	0.37	0.26	0.03
2.2	0.5	0.37	0.11
2.4	0.39	0.73	0.2
2.6	0.35	0.24	0.05
2.8	0.43	0.14	0.03

To assess the performance of our estimator *Ŷ*_BC_, we bin the data of *Y*_test_*vsŶ*_BC_, according to the values of *Ŷ*_BC_, with a bin width of 0.5 in the log scale, and represent the statistics of *Y*_test_ for each bin in box plots in Figures 7B, S9B, S10B, and S11B, for the various drive values tested. The data are compared to a linear line with a unit slope (*y* = *Y*_test_), which represents the results of a perfect predictor. For low drive values (Figures 7B and S9B), the predictor agrees well with the true values, whereas for high drive values (Figures S10B and S11B), the predictor agrees well mostly for intermediate values of *Ŷ*_BC_, probably due to insufficient statistics for high and low values of *Ŷ*_BC_.

To compare the mean error of the predicted values *Ŷ*_BC_ with the mean value of *Ŷ*_M_, the absolute values of error distributions for each bin, Δ*Ŷ*_BC_ and Δ*Ŷ*_M_, are depicted in box plots in Figures 7C, S9C, S10C, and S11C for the different drive values. The relative number of data points in each bin appears as a colored circle above the corresponding boxes. Indeed, the results of the SLM outperform the naïve mean predictor manifested in the smaller absolute mean values of Δ*Ŷ*_BC_ compared to Δ*Ŷ*_M_. As excepted, the prediction is more accurate for shorter remaining times, following the intuition that when the system is closer to the global minimum, it may be easier to predict its future behavior. Close to the assembled state, when there is a relatively large assembled seed, the system is more likely to assemble the target rather than disassemble to the individual components owing to the energy barrier. For all simulated drive values, we found that KLD_BC_ < KLD_M_ (Table 2), indicating the advantage of *Ŷ*_BC_ compared to *Ŷ*_M_.

In order to validate the robustness of the SLM approach, we performed a sensitivity analysis by changing the simulation parameters, one factor at a time.^115^ Specifically, the number of particles was increased to *N* = 36 while keeping the number of stored targets *M*_T_ = 2, whereas, in another set of simulations, the number of stored targets was varied between *M*_T_ = 1, 3, 4 while keeping the initial choice of the number of particles *N* = 25. The rest of the simulation parameters were chosen according to the values in Table 1. We simulated the system with 1000 realizations for each drive value and activated the SLM (Figure 6), considering only drive values for which more than 60% of the trajectories contained at least one assembly event for the prediction, and calculated the corresponding Pearson correlation coefficients. Moreover, we compared the KLD_M_ with the KLD_BC_ results to ensure the SLM provided predictive information (see Sensitivity Analysis in the Supporting Information).

For both the variation in the number of particles (Figure S13 and Table S3) and the number of stored targets (Figures S14–S16 and Tables S4–S6), we found that *R* > 0.25 for all of the simulated system configurations, indicating the correlation between the true and predicted values of the SLM. Furthermore, for all 39 simulated system configurations, with the exception of 3 cases, we found that KLD_BC_ < KLD_M_, demonstrating the improved prediction of the SLM compared to the naïve guess of the median value of the remaining time to the first self-assembly. These results further support the predictive power and robustness of the SLM approach.

## Conclusions

This work quantitatively explores nonequilibrium driving in self-assembly processes. We have used a toy model of interacting distinguishable particles that can assemble target structures, which are global energy minima of the system. We found that the time to the first assembly had a log-normal distribution for most drives simulated, whose median and standard deviation decreased exponentially with increasing the drive value. Moreover, we have established a novel data-driven general framework, the stochastic landscape method (SLM), for inferring the stochastic dynamics and providing predictions. Here, we used the SLM to provide a prediction for the remaining time to the first assembly, given the statistics of a down-sampled observable. We further showed that the SLM holds predictive power for different parameter values of the system, demonstrating its robustness.

Our method was theoretically inspired by the prevalence of Kramer’s escape rate in nonequilibrium biological systems,^116^ and the concept of “low rattling,”^48^ stating that a system tends to find regions in phase space in which fluctuations are small, making it less likely for it to escape. Another inspiration was drawn from dynamical systems theory, where systems are studied from a top-down perspective.^80,81^ Our approach utilizes a statistics-based segmentation algorithm^89^ for constructing the phase space of the system^79^ from a trajectory observable,^80,81^ in order to provide predictions. We have shown that using different measures of prediction evaluation, the SLM yields better results compared to the trivial guess for the remaining time to self-assembly, where the prediction is more accurate for shorter times since the system is closer to the global minimum.

Although predictability is challenging for stochastic systems,^117,118^ the resulting correlation values between the measured and predicted values of the remaining time to the first assembly manifest a predictive power of the SLM. Inevitably, in some cases, the SLM prediction is limited, as can be seen in Figure 7, for larger times. For example, if the system is randomly initiated, the *t*_*r*_ prediction can only be the initial broad distribution of *T*_FAS_ presented in Figure 3.

The SLM is a novel approach to study nonequilibrium stochastic dynamics with a rugged energy landscape, based on observing the statistical moments of an observable for a short duration. Based on the measured mean, standard deviation, and average trend of the observable, the SLM attributes a particular macrostate to the system, from which it can provide a prediction for the remaining time to the first assembly.

Future work will focus on finding stochastic differential equations^119,120^ for the segments representation of the system, assessing the prediction limit of the system, adding predictions based on segmented trajectories, examining the time complexity of the system for larger assemblies,^121^ utilizing the framework for real-time drive control protocols of the self-assembly process, allowing collective motions of clusters, using, for example, “virtual-move” Monte Carlo algorithms,^94,96^ and applying similar approaches of the SLM to other nonequilibrium systems, relying on its general predictive power. We envision that the SLM would promote a deeper understanding of biophysical nonequilibrium processes and advance the experimental realizations of synthetic self-assembly systems.

## Supporting Information Available

Simulation realization for equilibrium Δμ = 0 *K*_B_*T* (Movie S1) (AVI)
Simulation realization for Δμ = 1*K*_B_*T* (Movie S2) (AVI)
Simulation realization for Δμ = 2*K*_B_*T* (Movie S3) (AVI)
Model analysis; numerical implementation; distance from the target calculation; additional MC simulation results; The BEAST algorithm details; remaining time to fist assembly statistics; SLM implementation on nonequilibrium self-assembly; sensitivity analysis; and simulation equilibrium properties (Figure S1); macro-parameters trajectories for the drives of Δμ = 0, 1, and 2 *K*_B_*T* (Figure S2); additional simulation results statistics (Figure S3); simulation results of the remaining time to the first assembly statistics (Figure S4); typical energy trajectories *vs* time for the default simulation parameters and the results of BEAST segmentation (Figure S5); the stochastic landscape results for the primary predictor (Figure S6); the bias correction scheme (Figure S7); the probability density functions of the bin-wise errors for Δμ = 1.6 *K*_B_*T* (Figure S8); results of the SLM prediction values for Δμ= 1 *K*_B_*T* (Figure S9); results of the SLM prediction values for Δμ = 2.2 *K*_B_*T* (Figure S10); results of the SLM prediction values for Δμ = 2.8 *K*_*B*_*T* (Figure S11); results of the SLM prediction values for Δμ = 2.6 *K*_B_*T* (Figure S12); results of the SLM prediction values for Δμ = 2.6 *K*_B_*T* for *N* = 36 particles (Figure S13); results of the SLM prediction values for Δμ = 2.6 *K*_B_*T* for *M*_*T*_ = 1 stored targets (Figure S14); results of the SLM prediction values for Δμ = 2.6 *K*_B_*T* for *M*_*T*_ = 3 stored targets (Figure S15); results of the SLM prediction values for Δμ = 2.6 *K*_*B*_*T* for *M*_*T*_ = 4 stored targets (Figure S16); median fit parameters for *T*_fas_(Δμ) (Table S1); STD fit parameters for *T*_fas_(Δμ) (Table S2); performance evaluation of the SLM for *M*_T_ = 2 and *N* = 36 (Table S3); performance evaluation of the SLM for *M*_T_ = 1 and *N* = 25 (Table S4); performance evaluation of the SLM for *M*_T_ = 3 and *N* = 25 (Table S5); performance evaluation of the SLM for *M*_T_ = 4 and N = 25 (Table S6) (PDF)

## Supplementary Material

jp3c01376_si_001.avi

jp3c01376_si_002.avi

jp3c01376_si_003.avi
