# Supplementary material for: Nonequilibrium Self-Assembly Time Forecasting by the Stochastic Landscape Method
Source: J Phys Chem B. 2023 Jul 5;127(27):6113–24. doi: 10.1021/acs.jpcb.3c01376 (PMC10350916; doi:10.1021/acs.jpcb.3c01376)
Supplement: Supplementary file 4 — jp3c01376_si_004.pdf [file jp3c01376_si_004.pdf]

Supporting Information: Nonequilibrium Self Assembly Time Forecasting by the Stochastic Landscape Method

Michael Faran† and Gili Bisker*,†,‡,¶,§

†*Department of Biomedical Engineering, Faculty of Engineering, Tel Aviv University, Tel Aviv 69978, Israel*

‡*The Center for Physics and Chemistry of Living Systems, Tel Aviv University, Tel Aviv 6997801, Israel*

¶*The Center for Nanoscience and Nanotechnology, Tel Aviv University, Tel Aviv 6997801, Israel*

§*The Center for Light-Matter Interaction, Tel Aviv University, Tel Aviv 6997801, Israel*

E-mail: bisker@tauex.tau.ac.il

Model Analysis

We begin by analyzing the performance of the nonequilibrium self-assembly system presented in the main text, quantified by time to first assembly, T_{FAS}, and the total time spent at the target state, T_{target}, given the simulation was initiated in one of the stored targets.

In contrast to the previous work,[1] here, the target can be assembled anywhere on the lattice rather than only in the center, the definition of the target is based on the adjacency matrix rather than the normalized Hamming distance, the inner states of the particles at the beginning of the simulation are the same rather than randomized, and the boundary conditions are periodic rather than repulsive.

The target structure is set to a 5×5 square,[2–4] so the number of particles is $N = 25$, providing us the opportunity to encode several targets that are different from each other. Larger systems, on the other hand, would require longer computation times, and limit our ability to thoroughly explore a larger parameter space. The grid size, $L \times L$, is chosen as $L = 15$, to allow for the particles to assemble the target in a reasonable simulation time, without crowding the system.

The total simulation time, T, is based on the time scale set by T_{FAS}, while the $J_w = 1$ is an arbitrary choice that satisfies $|J_w| < |J_s|$ for all the simulated J_s values. To examine the effects of interactions strength and encoded targets number on T_{FAS}, the strong interaction J_s is varied in the range between -7.5 K_BT and -3 K_BT, and the number of targets, M_T, is varied between 1 and 4.

The results for T_{FAS} and T_{Target} as a function of J_s for $M_T = 1, 2, 3, 4$ are depicted in Fig. S1, showing the fundamental trade-off in equilibrium self-assembly, where target stability, quantified by T_{target}, can come at the cost of the assembly speed, quantified by T_{FAS}. Clearly, larger absolute values of J_s result in a more stable assembly, *i.e.*, higher T_{target}. However, the time to first assembly, T_{FAS}, does not show a monotonic trend. For low absolute values of J_s, the target cannot be assembled as the interaction is too weak, and high absolute values of J_s result in kinetic traps that hinder the assembly. Instead, the optimal

T_{FAS} is found in an intermediate value of J_s, in which the target stability is compromised. Moreover, this optimal J_s value varies with the number of stored targets, M_T. This behavior aligns with previous findings.[1]

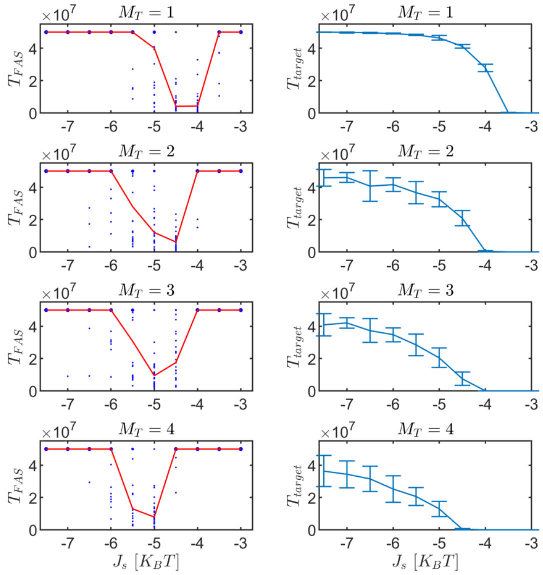

Figure S1: Equilibrium simulation results for different numbers of stored targets, $M_T = 1, 2, 3, 4$ (top to bottom row, respectively). (A) The median of the time to the first self-assembly, T_{FAS}, as a function of the strong interaction, J_s. Blue dots represent the results of individual realizations, where larger markers stand for multiple realizations resulting in the same value. The median value, depicted in a red line, is calculated for 24 realization for each value of J_s. (B) The mean time spent at the target, T_{target}, for realizations initiated at one of the stored targets. Error bars stand for the standard deviation of 24 realization for each value of J_s.

Given the results in equilibrium, we chose to focus on the case of $M_T = 2$, which is the simplest scenario in which the particles switch between internal states, and the trade-off between target stability and assembly speed is apparent. In addition, the value for the strong interaction energy was chosen to be $J_s = -4\ K_B T$, as this is approximately the minimum absolute value of J_s in which assembly begins to occur, and the nonequilibrium drive could make a significant contribution.

Numerical Implementation

The numerical simulations were run using Python-based parallelized code. Cyclic boundary conditions were considered. The number of particles, grid size, and other parameters simulated are listed in Table 1. The initial locations of the particle were randomly chosen, while their initial internal state was set to $s_i = 1$.

Distance from Target Calculation

The distances from the targets, d_m, are calculated by observing the particles in the simulation as a uni-directed and unweighted graph representation.[5] The graph is first constructed separately for each of the stored targets $m = 1, \ldots, M_T$ (see Fig. 1C) and for the correct board state. The particles are the labeled nodes of the graph, the internal states of the particles are the node colors and the n.n. bonds between adjacent particles are the edges. The node “color” is set to $c(i) = n$ if the internal state of the particle i is n.

An $N \times N$ adjacency matrix is constructed for the graph as follows. First, all the matrix values are set to -1. Then, each row i represents the labeled particle i, and each column $j \neq i$ represents a bond with the labeled particle j. If an (i, j) bond exists in the graph, the (i, j) matrix element attains the value $R_u c(i)+c(j)$, where $R_u = 10^r$ and $r = \lceil \log_{10}(M_T) \rceil + 1$. This method guarantees a one-to-one mapping between the (i, j) bond and an integer. Similarly, the diagonal (i, i) elements attain the values $R_u c(i)+c(i)$. Consequently, non-diagonal matrix elements retain the initial value of -1 if and only if there is no n.n. bond formed between i and j.

The graphs that represent the target grids (see Fig. 1C) are denoted by G_m, and their respective adjacency matrices are A_m. The graph that represents the current state of the board (see Fig. 1A) is denoted by G_B with the adjacency matrix A_B.

Finally, d_m is calculated by counting all the non-zero (i, j) elements in the matrix $\Delta A = A_B - A_m$. If $A_m = A_B$, meaning all the particle bonds are identical to that of a target

structure, then $d_m = 0$, and the system has successfully assembled one of the stored targets. This unique distance definition under rotation, translation, and reflection symmetries.

Additional MC Simulation Results

This section presents additional trajectories of the energy, entropy production, and distance from the targets for drives values of $\Delta\mu = 0, 1, 2\ K_BT$ (Fig. S2), T_{FAS} statistics for the drive values $\Delta\mu = 0, 0.2, 2.8\ K_BT$ (Fig. S3), and fit parameters for the median and standard deviation (STD) vs. drive values (Table S1 and Table S2).

Simulation movies for drive values $\Delta\mu = 0, 1, 2\ K_BT$ are also available (Movie S1, Movie S2, and Movie S3).

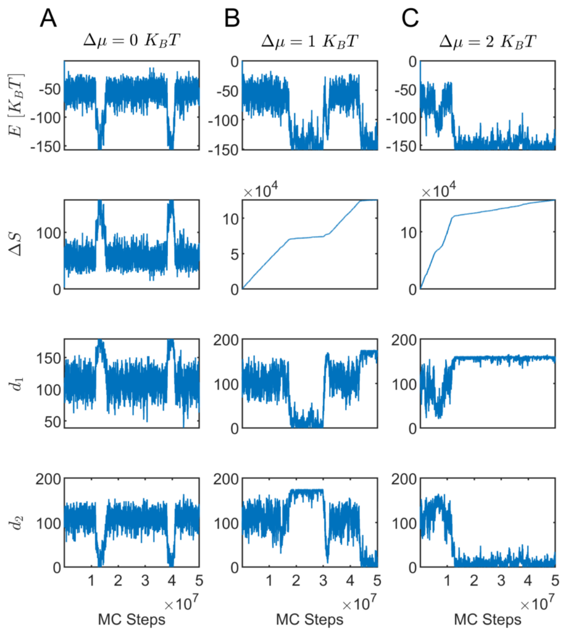

Figure S2: The energy E, total entropy production ΔS, and distance from the two targets d_1 and d_2 (from top to bottom row, respectively) vs. MC steps for the drive values (A) $\Delta\mu = 0\ K_BT$, (B) $\Delta\mu = 1\ K_BT$, (C) $\Delta\mu = 2\ K_BT$.

Table S1: Median fit parameters for $T_{FAS}(\Delta\mu)$

T_{FAS} *Median fit parameter*	Fitted Value	95% Confidence Intervals
A	8.3×10^7	$(7.7 \times 10^7, 8.8 \times 10^7)$
B	2.9	$(2.7, 3.2)$
C	2.5×10^6	$(1.75 \times 10^6, 3.2e \times 10^6)$

Table S2: STD fit parameters for $T_{FAS}(\Delta\mu)$

T_{fas} *STD fit parameter*	Fitted Value	Confidence Intervals (95%)
A	3.1×10^7	$(2.74 \times 10^7, 3.5 \times 10^7)$
B	2.2	$(1.8, 2.6)$
C	4.6×10^6	$(3.94 \times 10^6, 5.3 \times 10^6)$

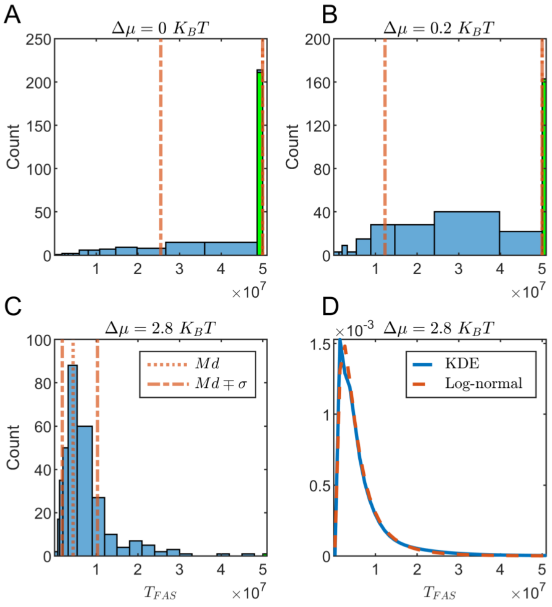

Figure S3: Additional simulation results statistics. (A) Histograms of the time to the first assembly for equilibrium $\Delta\mu = 0\ K_BT$ and (B) nonequilibrium drive value $\Delta\mu = 0.2\ K_BT$, and (C) nonequilibrium drive value $\Delta\mu = 2.8\ K_BT$. The dotted vertical lines are the median values, Md, of the histogram, and the dashed lines are the $16th$ and $84th$ percentiles of the data, respectively, corresponding to the standard deviation, σ. The green bins stand for the realizations in which no assembly occurred. (D) Fit for the simulation realization histograms of $\Delta\mu = 2.8\ K_BT$ with a kernel density estimator (KDE, blue), and a log-normal distribution (dashed orange).

Remaining Time to First Assembly Statistics

Here, we present the t_r histogram and fitted distributions for the drive values $\Delta\mu = 0.6, 1.2, 1.8, 2.4\ K_BT$ (Fig. S4A and Fig. S4B). For the t_r histograms, we used unequal binning to capture the underlying distribution of the data. For values below the median of the data, we chose equal binning in the log scale, whereas for values above the median, we used the standard Scott's rule for binning.[6] The data were fitted by a Kernel density estimator (KDE) with a positive support,[7] and a log-normal distribution. As the drive value increases, the fit of the log-normal distribution better captures the data.

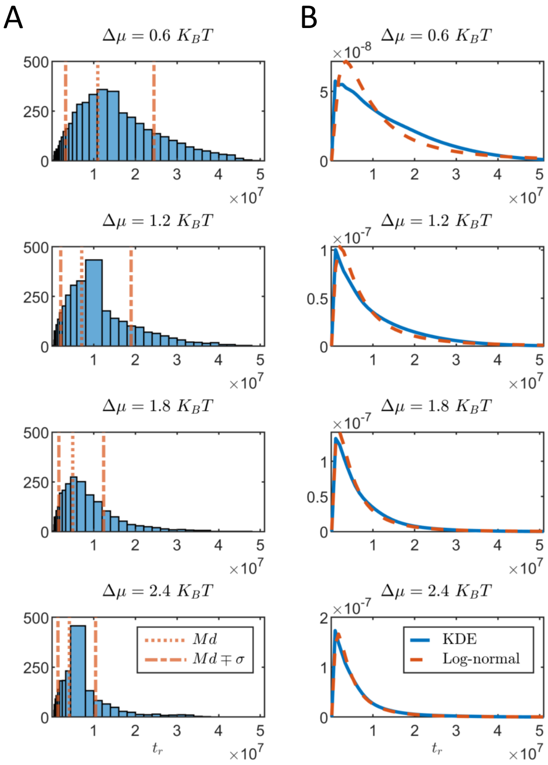

Figure S4: Simulation results of t_r statistics. (A) Simulation realizations histograms of the remaining time to the first assembly, for nonequilibrium drives, $\Delta\mu = 0.6$, 1.2, 1.8, and 2.4 K_BT. The dotted vertical lines are the median values, Md, of the histogram, and the dashed lines are the 16th and 84th percentiles of the data, respectively, corresponding to the standard deviation, σ. (B) Fit for the simulation realizations histograms with a kernel density estimator (KDE, blue), and a log-normal distribution (dashed orange).

The BEAST Algorithm Details

The BEAST algorithm assumes the given time series $y_i \in \vec{y}$ with samples $i = 1 \dots N_s$ is constructed from $m_s = 1, 2 \dots M_s$ different intervals with varying duration of τ_{s,m_s}. The variables m_s, τ_{s,m_s} are all together represented by a model vector $\vec{M}$. Each of the intervals is further characterized by trend and seasonal coefficients, represented by $\vec{\beta}_m$. A Gaussian noise $\epsilon = N(0, \sigma^2)$ is assumed for all the segments, where each segment has a different noise magnitude. The BEAST attempts to find the parameters $\vec{M}$, $\vec{\beta}_m$, and σ^2 that would yield the optimal trajectory segmentation using Bayesian inference. In other words, it chooses the parameters that maximize the posterior probability $P_{pr} = p(\vec{M}, \vec{\beta}_m, \sigma^2|\vec{y})$. P_{pr} attains an analytic form by first using the Bayes rule to decompose it into a product of the likelihood probability and prior probabilities. The likelihood probability is assumed to be a product of different Gaussian distributions due to the Gaussian nature of the noise, ϵ. The prior probabilities are based on the following assumptions: (1) A uniform prior distribution for all the values of $\vec{M}$ with a large cutoff, (2) A normal-inverse Gamma distribution along with a dispersion hyper-parameter to account for the $p(\vec{\beta}_m, \sigma^2|\vec{M})$ distribution. Although a closed-form expression is derived for P_{pr}, it is analytically intractable to find the optimal parameters. To overcome this, the BEAST uses a reverse jump Markov chain Monte Carlo (MCMC) sampler in a Gibbs sampling framework.[8] Eventually, the optimized parameters of $\vec{M}$, $\vec{\beta}_m$, and σ^2 are assessed by the algorithm.[9–11]

SLM Implementation on Nonequilibrium Self Assembly

The SLM aims to provide a forecast for the remaining time to self-assembly $Y = log(t_r)$. The SLM implementation follows the box diagram phases described in Fig. 6 in the main text.

Down-sampling the Data

Before implementing the BEAST algorithm on the raw data, we down-sample the energy trajectories data by a factor of 10^4 in order to abate the BEAST run-time. This results in 5×10^3 MC steps instead of the original 5×10^7 steps.

The BEAST Algorithm Implementation

The SLM method relies on the BEAST algorithm[9] for segmentation of the energy trajectories of the simulation realizations. After down-sampling, we chose a minimal segment duration as one percent of the total simulation time, to effectively limit the number of change points in the data. An example of the BEAST segmentation results is depicted in Fig. 5 in the main text, and Fig. S5.

We collect data of the energy E trajectories from 1000 realizations for each drive value, $\Delta\mu$. For each trajectory, l, we record the time to first assembly value, $T_{FAS,l}$, to be used as the data labels, where simulations in which no assembly occurs are labeled by the total trajectory length. The BEAST algorithm is implemented on all the trajectories, providing the segmentation. We enlist all the segments in a set denoted as Ω_E, and keep their origin trajectory number l and $T_{FAS,l}$. Moreover, for each segment, we record its initial MC step, t_{on}, in the original trajectory, as we are interested only in the segments that occurred before the first assembly.

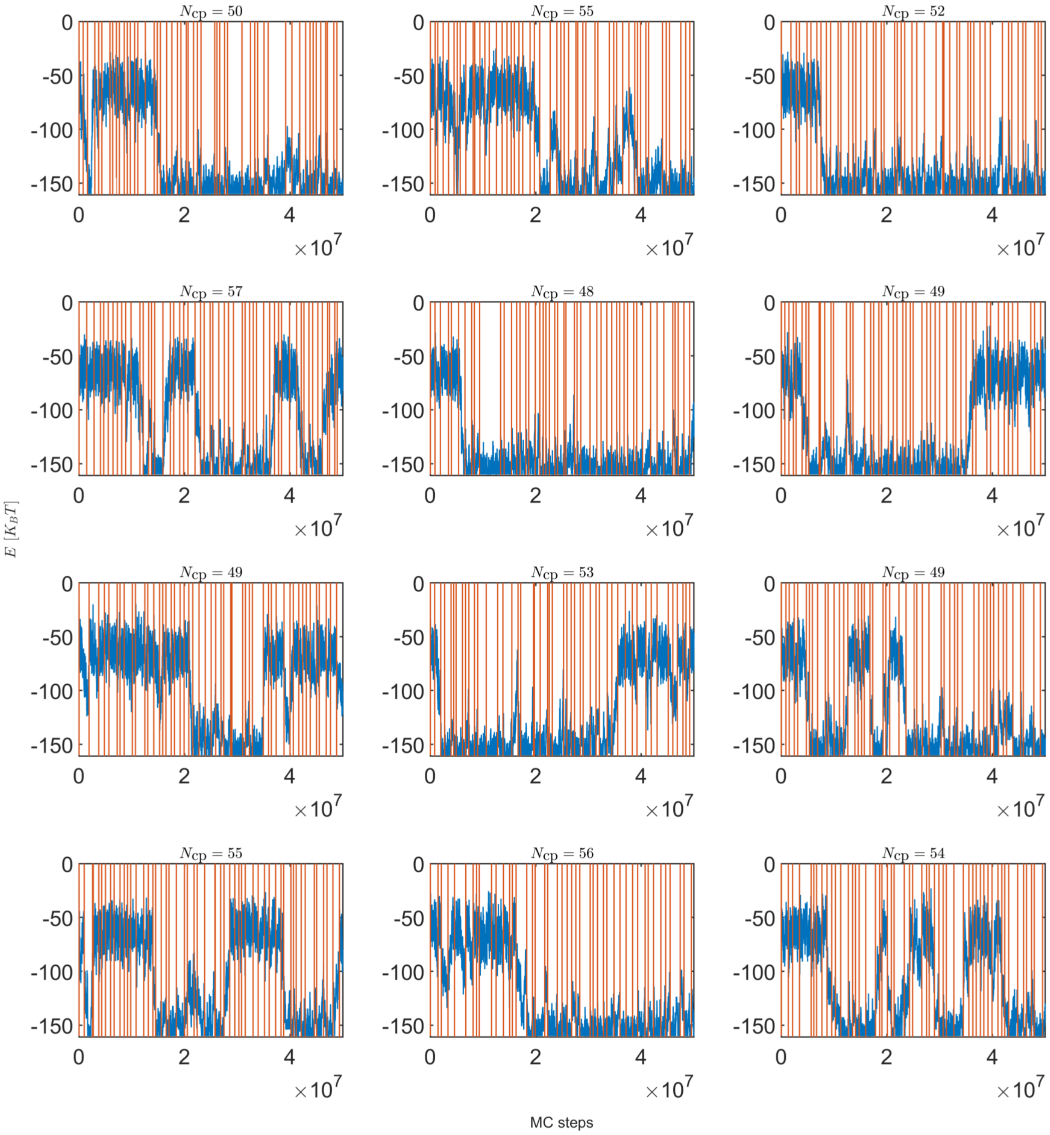

Figure S5: Typical energy trajectories vs. time for the default simulation parameters. The vertical lines indicate the change points in statistics detected by the BEAST algorithm. The titles of each panel are the number of change points detected, $N_{\rm cp}$. The parameters of the simulation appear in Table 1 in the main text, and the non-equilibrium drive is $\Delta\mu = 1\ K_BT$.

Stochastic Coordinates Data Extraction

After discarding the segments l whose initial MC step, t_{on}, is larger than $T_{FAS,l}$, we focus on the remaining set of segments, Ω_{FAS}, for training our model. The segments in Ω_{FAS} are ordered according to their sequential appearance in their original trajectory l, and are labeled with a running index $\alpha = 1 \ldots \Gamma_l$, where Γ_l in the total number of segments in l. Then, all the segments in Ω_{FAS}, indexed according to the trajectory and the segment number (l, α), are labeled by the remaining time to first assembly, $t_r(l, \alpha)$ calculated by the difference between $T_{FAS,l}$ and the corresponding t_{on} of the (l, α) segment (See "The Remaining Time to First Assembly" section in the main text).

We now assign each segment its stochastic coordinates. We take into account three statistical features for each segment (l, α): its mean energy $\mu_E(l, \alpha)$, its standard deviation $\sigma_E(l, \alpha)$, and its average trend $t_E(l, \alpha)$, so that each segment is characterized by $\{\mu_E, \sigma_E, t_E\}$, and its corresponding label, $Y = log(t_r)$, where for the sake of simplicity, we omit the indices (l, α). The stochastic coordinates are then normalized by subtracting their mean and dividing by their standard deviation, yielding $\{\mu_E^*, \sigma_E^*, t_E^*\}$, where * stands for the normalized coordinate. With this labeled data set at hand, we seek to unveil a mapping function $\hat{Y} = f(\mu_E^*, \sigma_E^*, t_E^*)$.

We first perform principal component analysis (PCA)[12] on the normalized coordinates and take the first two principal components, y_1 and y_2, so our target mapping is $\hat{Y} = g(y_1, y_2)$. For cross-validation (CV), we randomly divide the data into three sets, namely, the training set Ω_T, the CV set Ω_{CV} and the test set Ω_{test}, which correspond to 60%, 20%, and 20% of the data, respectively. The following steps are repeated 10 time: (1) Ω_T, and Ω_{CV} randomization, (2) Stochastic landscape construction, and (3) primary predictor establishment. The test set is left aside and does not take part in the CV process.

Randomization of the train and cross-validation sets

We randomize the segments found in the set $\Omega_{CV} \cup \Omega_T$ and redistribute them again into new sets of Ω_T and Ω_{CV}.

Stochastic Landscape Construction

We aim to generate a continuous function from the data in the training set, Ω_T, sampled from $Y = g(y_1, y_2)$. To this end, we use a standard 2-D triangulation, followed by a 2-D convolution with the arithmetic mean of a sliding window,[13] performed twice with windows sizes 2 and 7, consecutively. The values for y_1, y_2 coordinates outside the boundaries of the interpolated map were set to $T = 5 \times 10^7$.

The resulting continuous map is referred to as the Stochastic landscape and can be considered the primary predictor $\hat{Y}_p$ for any pair of values of y_1, y_2. An example of the stochastic landscape for a drive value of $\Delta\mu = 1.6\ K_BT$ is displayed in Fig. S6.

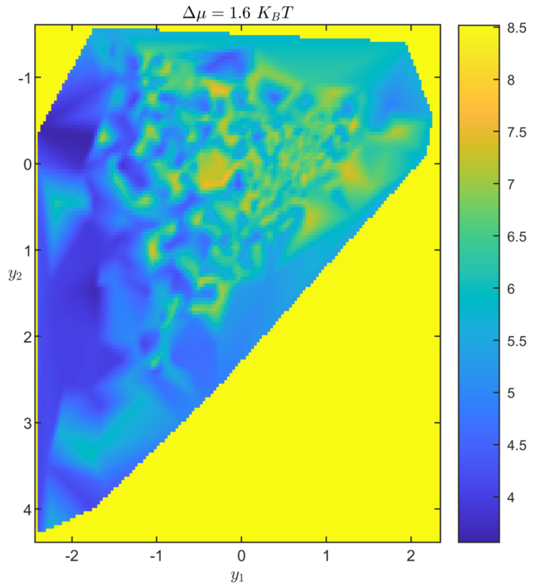

Figure S6: The stochastic landscape of the primary predictor $\hat{Y}_p$ for the remaining time to the first assembly based on the training set Ω_T. The color bar represents the $\hat{Y}_p$ values in a log scale. The two axes, y_1 and y_2 are the first two principal components of the PCA of the stochastic coordinates values for Ω_T.

The Primary Predictor Establishment

The values of the true values, Y, vs. the primary predictor $\hat{Y}_p$, are presented as a scatter plot in Fig. S7A, where each color represents a different CV iteration. The data is divided into bins with a width of 0.5 (in the units of the log of the remaining time to the first assembly), according to the $\hat{Y}_p$ value, and the mean value of Y for each CV iteration appears in Fig. S7B for each bin. Bins with less than 5 samples are discarded. The data is displayed with the primary predictor $\hat{Y}_p$ as the x-axis for easier visualization of the binning, which is done according to the $\hat{Y}_p$ values.

Averaging over all the CV iteration for the bins results in $\langle\hat{Y}_p\rangle_{CV}$, which is then compared to the linear line with a unit slope $y = Y$ representing the correct values.

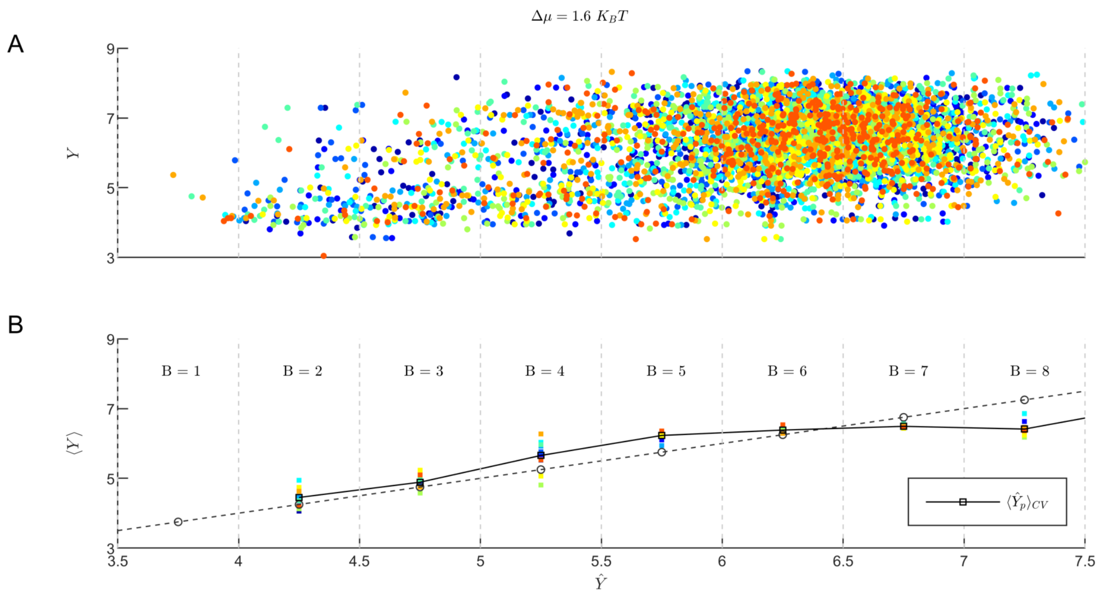

Figure S7: The bias correction scheme. (A) Scatter plots of the true values Y vs. the $\hat{Y}_p$ predictions for different cross-validation (CV) iterations, for drive value $\Delta\mu = 1.6\ K_BT$. The different colors stand for different iteration results. The dashed vertical lines indicate the bin separation. (B) The scatter plots average $\langle Y\rangle$ for each bin vs. the center of the $\hat{Y}_p$ bin, where the different colors represent different CV iterations. The mean $\hat{Y}_p$ values for all the CV iterations, $\langle\hat{Y}_p\rangle_{CV}$, appear as the black squared, and they are compared to a perfect predictor represented by a black dashed line, $y = Y$. The bin number is written above each data point.

The SLM Predictor

We seek to correct the bias of the $\langle \hat{Y}_p \rangle_{CV}$ values, compared to the true values, Y (Fig. S7B), in order to provide a more accurate prediction for Ω_{test} based on the data in $\Omega_T \cup \Omega_{CV}$. To this end, we follow the following steps: (1) We construct the stochastic landscape for $\Omega_T \cup \Omega_{CV}$, and establish the corresponding primary predictor $\hat{Y}_p$. (2) We use $\hat{Y}_p$ to provide predictions for the Y data of Ω_{test}. (3) The values of $\hat{Y}_p$ vs. Y_{test} are depicted as grey dots in the scatter plot in Fig. 7A, where Y_{test} are the true values of Ω_{test}. (4) The data are binned according to the $\hat{Y}_p$ values, similar to the primary predictor step. (5) The bias in the prediction is calculated by the difference between the bin-wise mean of the $\hat{Y}_p$ data, $\langle \hat{Y}_p \rangle_{CV}$, and the bin-wise mean of the Y_{test} data, where a positive difference means an overestimation. (6) The bias is subtracted from the $\hat{Y}_p$ values in the corresponding bin, resulting in the biased corrected SLM predictor, $\hat{Y}_{BC}$ (black squares in Fig. 7A). Subsequently, the bin-wise probability distributions of $\Delta\hat{Y}$ are calculated (Fig. S8) for evaluating the SLM performance.

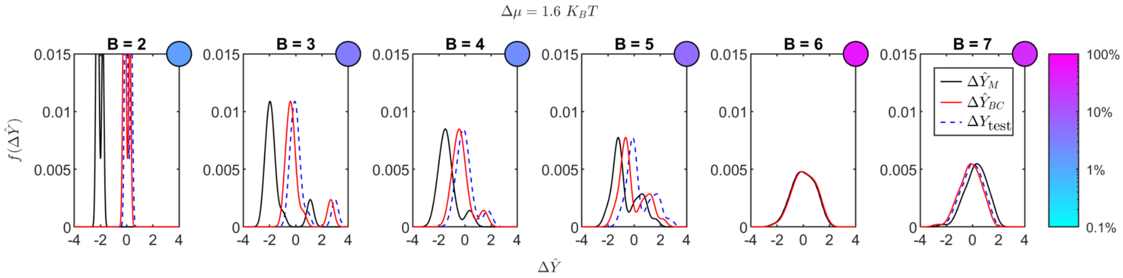

Figure S8: The bin-wise probability distribution of the data ΔY_{test} (dashed blue), the mean predictor $\Delta\hat{Y}_M$ (black), and the SLM predictor $\Delta\hat{Y}_{BC}$ (red), for $\Delta\mu = 1.6\ K_BT$. The bin numbers are the same as in Fig. S7. The color bar indicates the relative weight of the data for each bin, calculated from the relative number of data points.

The SLM Output Presentation

The true values, Y_{test}, are depicted vs. the bias corrected SLM predictor values, $\hat{Y}_{BC}$, for drive values $\Delta\mu = 1, 1.6, 2.2, 2.8\ K_BT$ in Fig. S9A, Fig. 7A (main text), Fig. S10A, and Fig. S11A respectively, and are depicted as black squares.

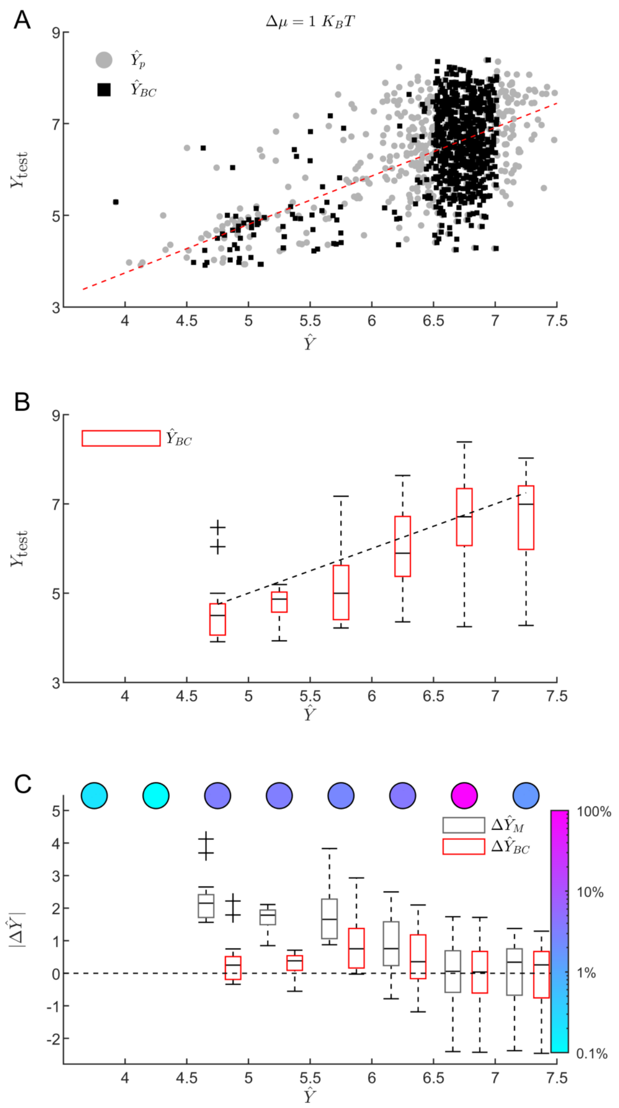

Figure S9: Results of the SLM prediction for the remaining time to the first assembly for the test set, for $\Delta\mu = 1\ K_{\mathrm{B}}T$ (A) The Y_{test} results are shown vs. their predicted values $\hat{Y}$, before the bias correction ($\hat{Y}_p$, grey circles), and after the bias correction ($\hat{Y}_{BC}$, black squares). The red line shows the linear regression of the data. (B) A box plot of the Y_{test} vs. the calculated $\hat{Y}_{BC}$, where the data is binned according to the $\hat{Y}_{BC}$ values (see main text). The box-plot whiskers represent the standard interquartile range (IQR), and outliers are defined to be above 1.5 IQR (plus markers). The dashed unit slope, linear line ($y = Y_{\text{test}}$) represents the results of a perfect predictor. (C) The absolute values of $\Delta\hat{Y}_M$ (black) and $\Delta\hat{Y}_{BC}$ (red) vs. the respective predictor value $\hat{Y}$ for each bin. The dashed black line at $\Delta\hat{Y} = 0$ represents a perfect predictor with zero error. The color bar indicates the relative weight of the data for each bin, calculated from the relative number of data points. Bins with weights less than 1% are omitted.

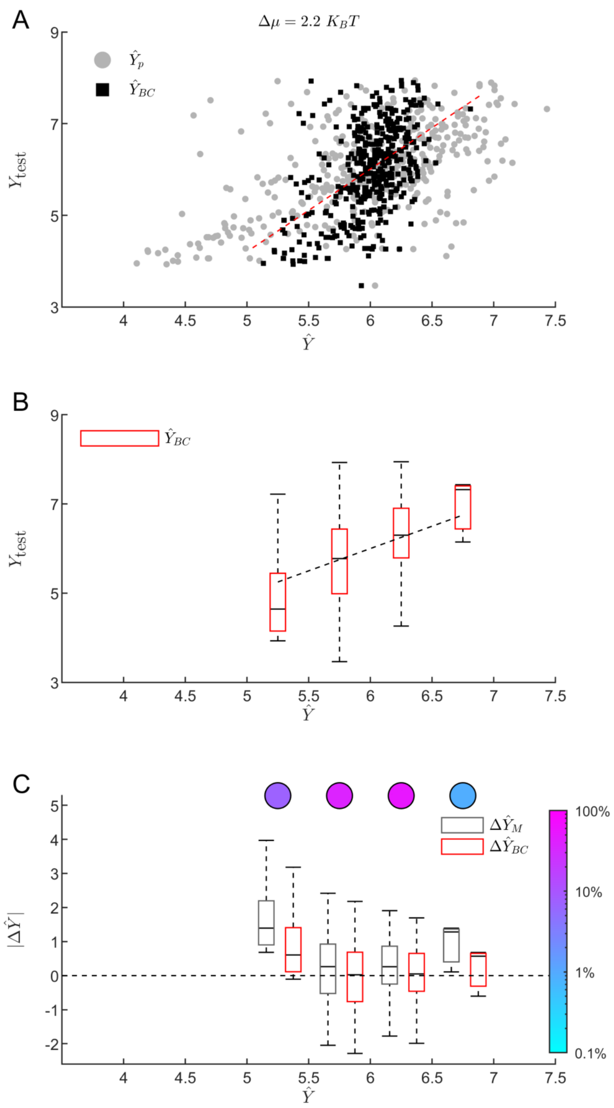

Figure S10: Results of the SLM prediction for the remaining time to the first assembly for the test set, for $\Delta\mu = 2.2\ K_{\mathrm{B}}T$ (A) The Y_{test} results are shown vs. their predicted values $\hat{Y}$, before the bias correction ($\hat{Y}_p$, grey circles), and after the bias correction ($\hat{Y}_{BC}$, black squares). The red line shows the linear regression of the data. (B) A box plot of the Y_{test} vs. the calculated $\hat{Y}_{BC}$, where the data are binned according to the $\hat{Y}_{BC}$ values (see main text). The box-plot whiskers represent the IQR, and outliers are defined to be above 1.5 IQR (plus markers). The dashed unit slope, linear line ($y = Y_{\text{test}}$) represents the results of a perfect predictor. (C) The absolute values of $\Delta\hat{Y}_M$ (black) and $\Delta\hat{Y}_{BC}$ (red) vs. the respective predictor value $\hat{Y}$ for each bin. The dashed black line at $\Delta\hat{Y} = 0$ represents a perfect predictor with zero error. The color bar indicates the relative weight of the data for each bin, calculated from the relative number of data points. Bins with weights less than 1% are omitted.

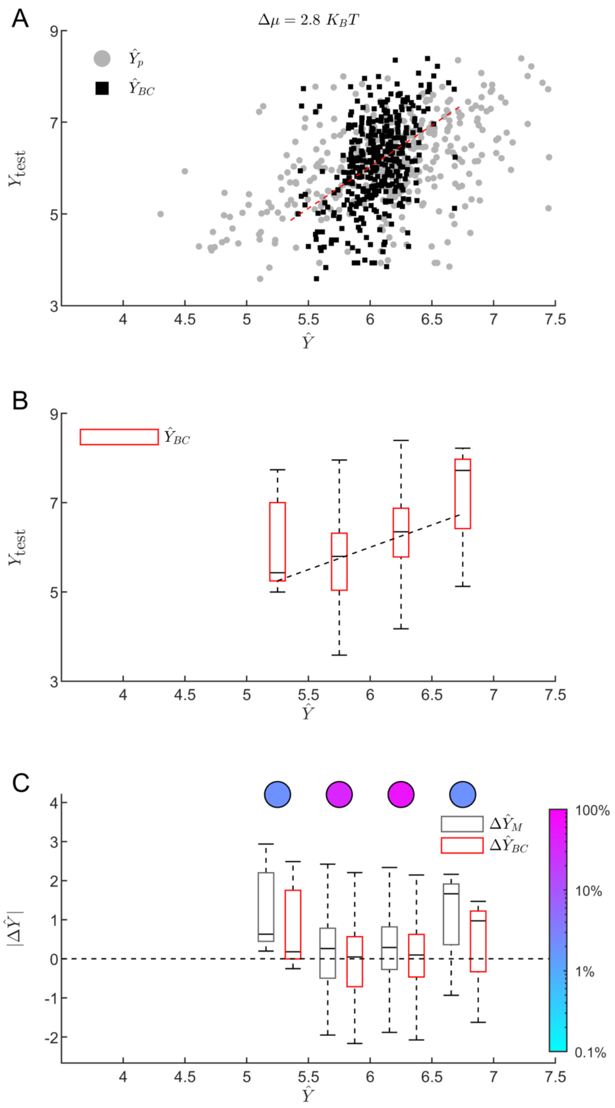

Figure S11: Results of the SLM prediction for the remaining time to the first assembly for the test set, for $\Delta\mu = 2.8\ K_\mathrm{B}T$ (A) The Y_test results are shown vs. their predicted values $\hat{Y}$, before the bias correction ($\hat{Y}_p$, grey circles), and after the bias correction ($\hat{Y}_{BC}$, black squares). The red line shows the linear regression of the data. (B) A box plot of the Y_test vs. the calculated $\hat{Y}_{BC}$, where the data are binned according to the $\hat{Y}_{BC}$ values (see main text). The box-plot whiskers represent the IQR, and outliers are defined to be above 1.5 IQR (plus markers). The dashed unit slope, linear line ($y = Y_\text{test}$) represents the results of a perfect predictor. (C) The absolute values of $\Delta\hat{Y}_M$ (black) and $\Delta\hat{Y}_{BC}$ (red) vs. the respective predictor value $\hat{Y}$ for each bin. The dashed black line at $\Delta\hat{Y} = 0$ represents a perfect predictor with zero error. The color bar indicates the relative weight of the data for each bin, calculated from the relative number of data points. Bins with weights less than 1% are omitted.

Sensitivity Analysis

We have tested the performance of the SLM approach for different system parameters, changing a single parameter at a time, while the rest of the parameters remained as listed in Table 1. For performance evaluation, we calculated the Pearson correlation coefficient, R, and compared the KLD_M to the KLD_{BC} values (see main text).

First, we increased the number of particles to $N = 36$ (Table S3). Then, we changed the number of stored targets to $M_T = 1$ (Table S4), $M_T = 3$ (Table S5), and $M_T = 4$ (Table S6), while keeping the original number of particles, $N = 25$. The full SLM results are given here for the comparison between its performance for the original parameter set (Fig. S12), with the performance for $N = 36$ (Fig. S13), $M_T = 1$ (Fig. S14), $M_T = 3$ (Fig. S15), and $M_T = 4$ (Fig. S16), all for the drive value $\Delta\mu = 2.6\ K_{\mathrm{B}}T$.

For all simulated parameters, $R > 0$, indicating the predictive power of the SLM approach. Moreover, with only three exceptions out of 39 simulation sets with different drive values, $KLD_{BC} < KLD_M$. Hence, the SLM framework indeed provides better prediction compared to the naïve guess of the median value for almost all the different systems tested.

Table S3: Performance evaluation of the SLM for $M_T = 2$ and $N = 36$

$\Delta\mu$	R	KLD_M	KLD_{BC}
0.6	0.56	0.98	0.35
0.8	0.56	1.0	0.07
1.0	0.59	0.61	0.08
1.2	0.55	0.58	0.14
1.4	0.67	1.02	0.21
1.6	0.55	1.06	0.21
1.8	0.52	0.36	0.14
2.0	0.53	0.6	0.19
2.2	0.34	0.41	0.32
2.4	0.44	0.56	0.29
2.6	0.4	0.58	0.11
2.8	0.46	0.29	0.04

Table S4: Performance evaluation of the SLM for $M_T = 1$ and $N = 25$

$\Delta\mu$	R	KLD_M	KLD_{BC}
0.6	0.54	0.68	0.09
0.8	0.51	0.84	0.78
1.0	0.49	0.23	0.23
1.2	0.38	0.43	0.15
1.4	0.45	0.24	0.03
1.6	0.42	0.21	0.04
1.8	0.42	0.14	0.02
2.0	0.48	0.97	0.22
2.2	0.44	0.26	0.06
2.4	0.42	0.32	0.09
2.6	0.38	0.35	0.08
2.8	0.28	0.42	0.12

Table S5: Performance Evaluation of the SLM for $M_T = 3$ and $N = 25$

$\Delta\mu$	R	KLD_M	KLD_{BC}
1.2	0.42	0.01	0.02
1.4	0.34	0.1	0.05
1.6	0.46	0.76	0.08
1.8	0.52	0.99	0.24
2.0	0.59	1.26	0.62
2.2	0.49	1.05	0.37
2.4	0.56	0.1	0.05
2.6	0.55	1.82	0.74
2.8	0.44	1.14	0.67

Table S6: Performance Evaluation of the SLM for $M_T = 4$ and $N = 25$

$\Delta\mu$	R	KLD_M	KLD_{BC}
1.8	0.39	0.53	0.05
2.0	0.41	0.5	0.1
2.2	0.38	0.01	0.09
2.4	0.39	0.64	0.19
2.6	0.42	0.4	0.04
2.8	0.26	0.01	0.06

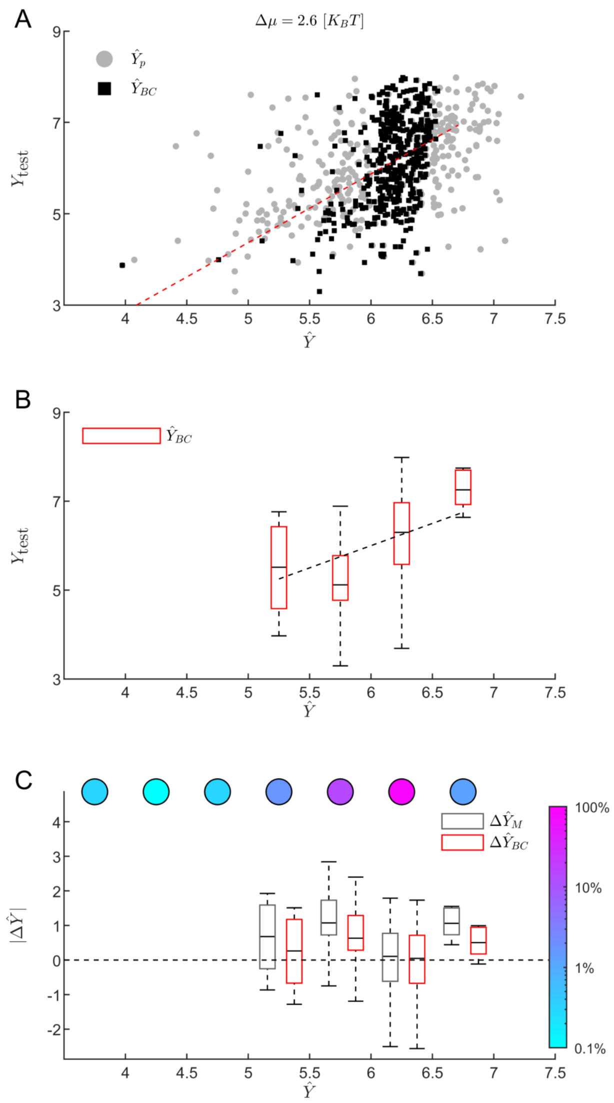

Figure S12: Results of the SLM prediction for the remaining time to the first assembly for the test set, for $\Delta\mu = 2.6\ K_\mathrm{B}T$ with the simulation default parameters. (A) The Y_test results are shown vs. their predicted values $\hat{Y}$, before the bias correction ($\hat{Y}_p$, grey circles), and after the bias correction ($\hat{Y}_{BC}$, black squares). The red line shows the linear regression of the data. (B) A box plot of the Y_test vs. the calculated $\hat{Y}_{BC}$, where the data are binned according to the $\hat{Y}_{BC}$ values (see main text). The box-plot whiskers represent the IQR, and outliers are defined to be above 1.5 IQR (plus markers). The dashed unit slope, linear line ($y = Y_\mathrm{test}$) represents the results of a perfect predictor. (C) The absolute values of $\Delta\hat{Y}_M$ (black) and $\Delta\hat{Y}_{BC}$ (red) vs. the respective predictor value $\hat{Y}$ for each bin. The dashed black line at $\Delta\hat{Y} = 0$ represents a perfect predictor with zero error. The color bar indicates the relative weight of the data for each bin, calculated from the relative number of data points. Bins with weights less than 1% are omitted.

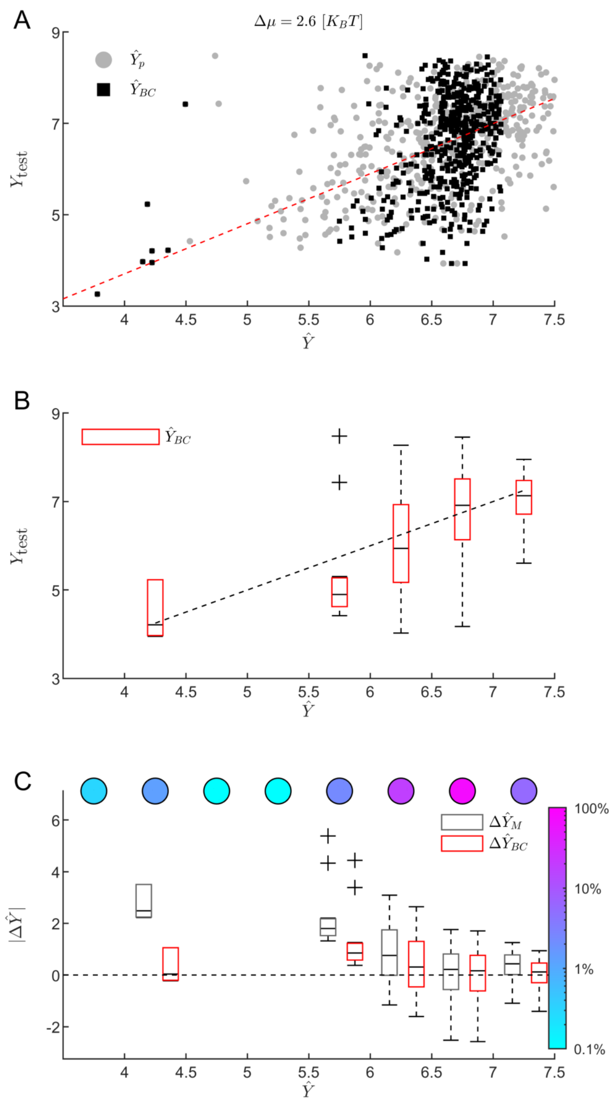

Figure S13: Results of the SLM prediction for the remaining time to the first assembly for the test set, for $\Delta\mu = 2.6\ K_BT$ and $N = 36$ particles. (A) The Y_{test} results are shown vs. their predicted values $\hat{Y}$, before the bias correction ($\hat{Y}_p$, grey circles), and after the bias correction ($\hat{Y}_{BC}$, black squares). The red line shows the linear regression of the data. (B) A box plot of the Y_{test} vs. the calculated $\hat{Y}_{BC}$, where the data are binned according to the $\hat{Y}_{BC}$ values (see main text). The box-plot whiskers represent the IQR, and outliers are defined to be above 1.5 IQR (plus markers). The dashed unit slope, linear line ($y = Y_{test}$) represents the results of a perfect predictor. (C) The absolute values of $\Delta\hat{Y}_M$ (black) and $\Delta\hat{Y}_{BC}$ (red) vs. the respective predictor value $\hat{Y}$ for each bin. The dashed black line at $\Delta\hat{Y} = 0$ represents a perfect predictor with zero error. The color bar indicates the relative weight of the data for each bin, calculated from the relative number of data points. Bins with weights less than 1% are omitted.

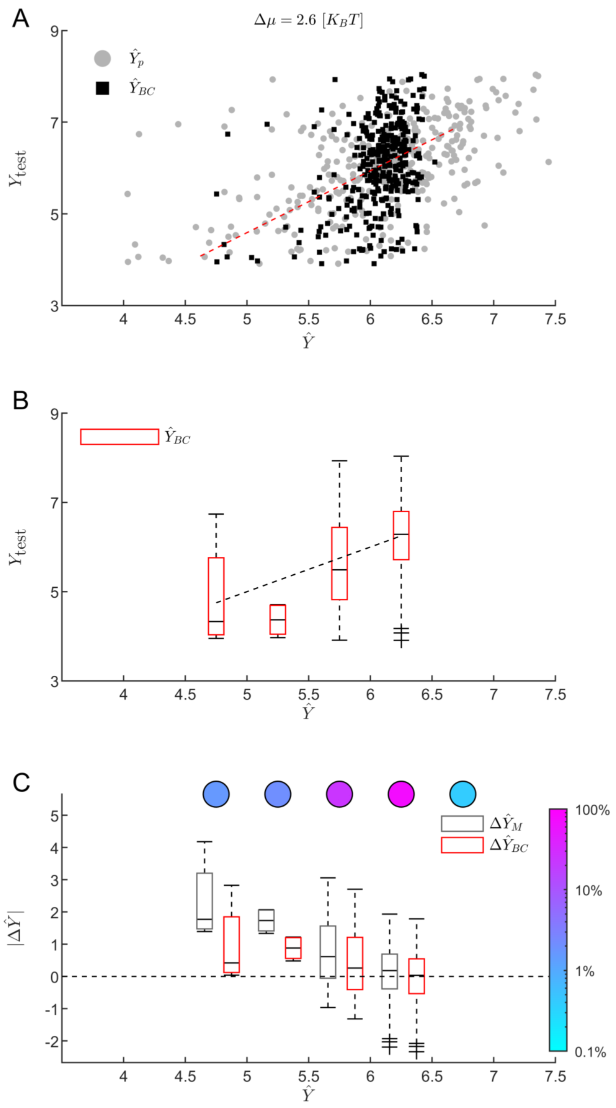

Figure S14: Results of the SLM prediction for the remaining time to the first assembly for the test set, for $\Delta\mu = 2.6\ K_{\mathrm{B}}T$ and $M_T = 1$ stored target. (A) The Y_{test} results are shown vs. their predicted values $\hat{Y}$, before the bias correction ($\hat{Y}_p$, grey circles), and after the bias correction ($\hat{Y}_{BC}$, black squares). The red line shows the linear regression of the data. (B) A box plot of the Y_{test} vs. the calculated $\hat{Y}_{BC}$, where the data are binned according to the $\hat{Y}_{BC}$ values (see main text). The box-plot whiskers represent the IQR, and outliers are defined to be above 1.5 IQR (plus markers). The dashed unit slope, linear line ($y = Y_{\text{test}}$) represents the results of a perfect predictor. (C) The absolute values of $\Delta\hat{Y}_M$ (black) and $\Delta\hat{Y}_{BC}$ (red) vs. the respective predictor value $\hat{Y}$ for each bin. The dashed black line at $\Delta\hat{Y} = 0$ represents a perfect predictor with zero error. The color bar indicates the relative weight of the data for each bin, calculated from the relative number of data points. Bins with weights less than 1% are omitted.

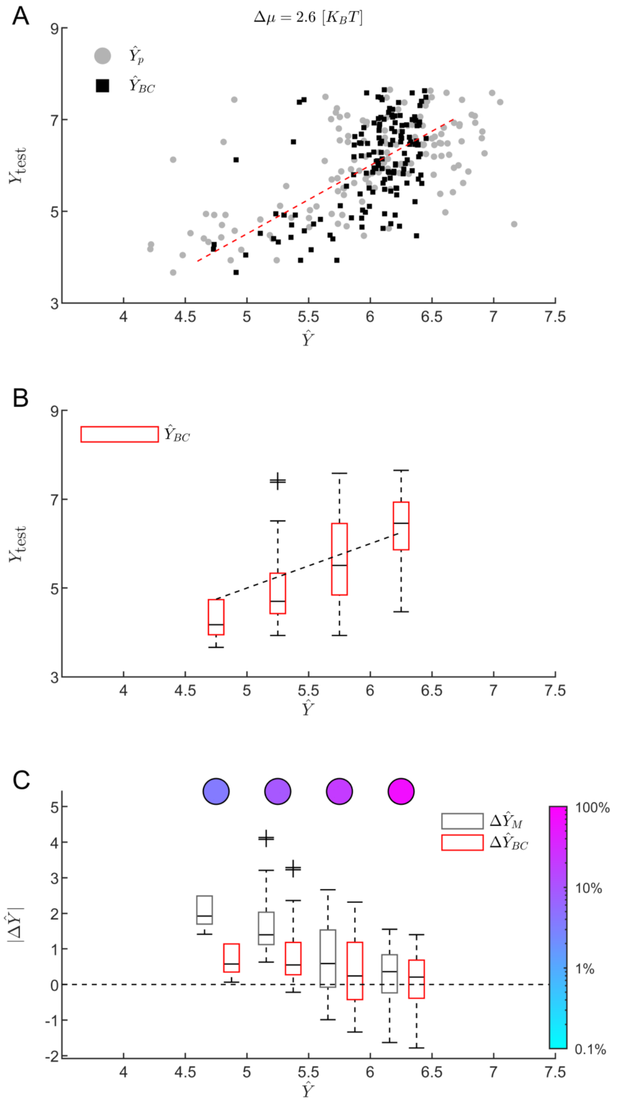

Figure S15: Results of the SLM prediction for the remaining time to the first assembly for the test set, for $\Delta\mu = 2.6\ K_BT$ and $M_T = 3$ stored target. (A) The Y_{test} results are shown vs. their predicted values $\hat{Y}$, before the bias correction ($\hat{Y}_p$, grey circles), and after the bias correction ($\hat{Y}_{BC}$, black squares). The red line shows the linear regression of the data. (B) A box plot of the Y_{test} vs. the calculated $\hat{Y}_{BC}$, where the data are binned according to the $\hat{Y}_{BC}$ values (see main text). The box-plot whiskers represent the IQR, and outliers are defined to be above 1.5 IQR (plus markers). The dashed unit slope, linear line ($y = Y_{\text{test}}$) represents the results of a perfect predictor. (C) The absolute values of $\Delta\hat{Y}_M$ (black) and $\Delta\hat{Y}_{BC}$ (red) vs. the respective predictor value $\hat{Y}$ for each bin. The dashed black line at $\Delta\hat{Y} = 0$ represents a perfect predictor with zero error. The color bar indicates the relative weight of the data for each bin, calculated from the relative number of data points. Bins with weights less than 1% are omitted.

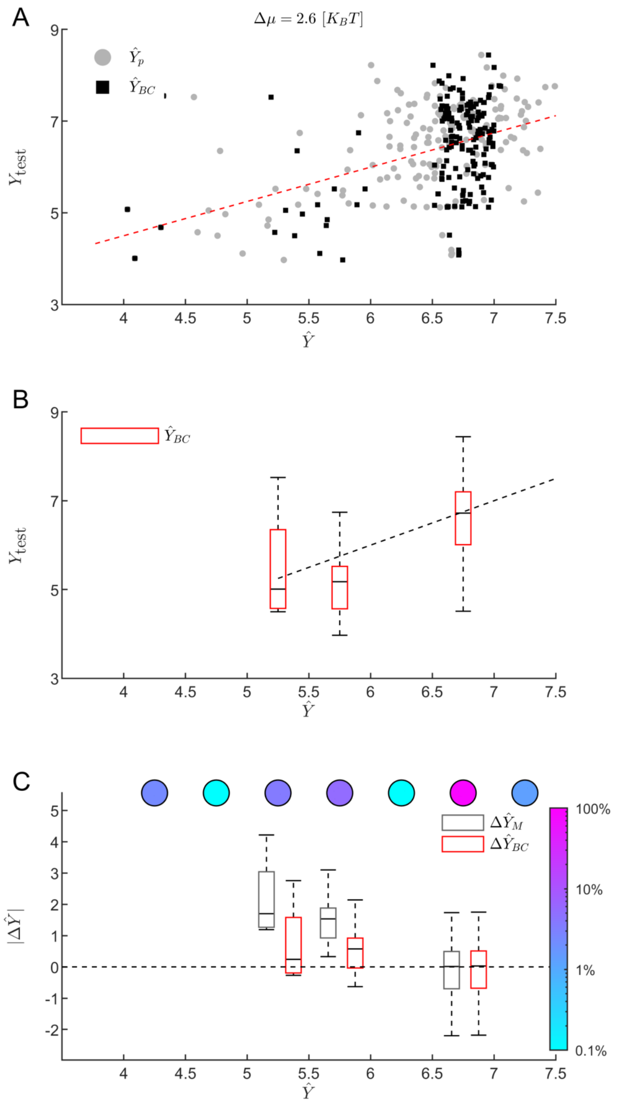

Figure S16: Results of the SLM prediction for the remaining time to the first assembly for the test set, for $\Delta\mu = 2.6\ K_\mathrm{B}T$ and $M_T = 4$ stored target. (A) The Y_{test} results are shown vs. their predicted values $\hat{Y}$, before the bias correction ($\hat{Y}_p$, grey circles), and after the bias correction ($\hat{Y}_{BC}$, black squares). The red line shows the linear regression of the data. (B) A box plot of the Y_{test} vs. the calculated $\hat{Y}_{BC}$, where the data are binned according to the $\hat{Y}_{BC}$ values (see main text). The box-plot whiskers represent the IQR, and outliers are defined to be above 1.5 IQR (plus markers). The dashed unit slope, linear line ($y = Y_{\text{test}}$) represents the results of a perfect predictor. (C) The absolute values of $\Delta\hat{Y}_M$ (black) and $\Delta\hat{Y}_{BC}$ (red) vs. the respective predictor value $\hat{Y}$ for each bin. The dashed black line at $\Delta\hat{Y} = 0$ represents a perfect predictor with zero error. The color bar indicates the relative weight of the data for each bin, calculated from the relative number of data points. Bins with weights less than 1% are omitted.

References

(1) Bisker, G.; England, J. L. Nonequilibrium associative retrieval of multiple stored self-assembly targets. *Proc. Natl. Acad. Sci. U. S. A.* **2018**, *115*, E10531–E10538.

(2) Ben-Ari, A.; Ben-Ari, L.; Bisker, G. Nonequilibrium self-assembly of multiple stored targets in a dimer-based system. *J. Chem. Phys.* **2021**, *155*, 234113.

(3) Osat, S.; Golestanian, R. Non-reciprocal multifarious self-organization. *Nat. Nanotechnol.* **2022**, 1–7.

(4) Gartner, F. M.; Graf, I. R.; Frey, E. The time complexity of self-assembly. *Proc. Natl. Acad. Sci. U. S. A.* **2022**, *119*, e2116373119.

(5) Asratian, A. S.; Denley, T. M.; Häggkvist, R. *Bipartite graphs and their applications*; Cambridge University Press: Cambridge, U.K., 1998; Vol. 131.

(6) Knuth, K. H. Optimal data-based binning for histograms. *arXiv preprint physics/0605197* **2006**, `https://arxiv.org/abs/physics/0605197` (accessed 2023-05-07).

(7) Bowman, A. W.; Azzalini, A. *Applied smoothing techniques for data analysis: the kernel approach with S-Plus illustrations*; Oxford University Press: Oxford, U.K., 1997; Vol. 18.

(8) Zhao, K.; Valle, D.; Popescu, S.; Zhang, X.; Mallick, B. Hyperspectral remote sensing of plant biochemistry using Bayesian model averaging with variable and band selection. *Remote Sen. Environ.* **2013**, *132*, 102–119.

(9) Zhao, K.; Wulder, M. A.; Hu, T.; Bright, R.; Wu, Q.; Qin, H.; Li, Y.; Toman, E.; Mallick, B.; Zhang, X., et al. Detecting change-point, trend, and seasonality in satellite time series data to track abrupt changes and nonlinear dynamics: A Bayesian ensemble algorithm. *Remote Sen. Environ.* **2019**, *232*, 111181.

(10) Xu, Y.; Yu, L.; Li, W.; Ciais, P.; Cheng, Y.; Gong, P. Annual oil palm plantation maps in Malaysia and Indonesia from 2001 to 2016. *Earth Syst. Sci. Data* **2020**, *12*, 847–867.

(11) Yang, X.; Tian, S.; You, W.; Jiang, Z. Reconstruction of continuous GRACE/GRACE-FO terrestrial water storage anomalies based on time series decomposition. *J. Hydrol.* **2021**, *603*, 127018.

(12) Abdi, H.; Williams, L. J. Principal component analysis. *Wiley Interdiscip. Rev. Comput. Stat.* **2010**, *2*, 433–459.

(13) Jain, P.; Tyagi, V. Spatial and frequency domain filters for restoration of noisy images. *IETE J. Educ.* **2013**, *54*, 108–116.
